# Supplementary material for: Qualitative study investigating the professional and personal effects of patient suicide on general practitioners in Northern Ireland
Source: BMJ Open. 2024 Feb 10;14(2):e077940. doi: 10.1136/bmjopen-2023-077940 (PMC10862281; doi:10.1136/bmjopen-2023-077940)
Supplement: Author's manuscript [file bmjopen-2023-077940.draft_revisions.pdf]

BMJ Open

BMJ Open is committed to open peer review. As part of this commitment we make the peer review history of every article we publish publicly available.

When an article is published we post the peer reviewers' comments and the authors' responses online. We also post the versions of the paper that were used during peer review. These are the versions that the peer review comments apply to.

The versions of the paper that follow are the versions that were submitted during the peer review process. They are not the versions of record or the final published versions. They should not be cited or distributed as the published version of this manuscript.

BMJ Open is an open access journal and the full, final, typeset and author-corrected version of record of the manuscript is available on our site with no access controls, subscription charges or pay-per-view fees (http://bmjopen.bmj.com).

If you have any questions on BMJ Open's open peer review process please email info.bmjopen@bmj.com

BMJ Open

General Practitioners and patient suicide: A qualitative study investigating the professional and personal effects.

Journal:	*BMJ Open*
Manuscript ID	bmjopen-2023-077940
Article Type:	Original research
Date Submitted by the Author:	19-Jul-2023
Complete List of Authors:	McAnee, Grainne; Ulster University, School of Psychology<br>Norwood, Kelly; Ulster University Faculty of Life and Health Sciences, School of Psychology<br>Leavey, Gerard; Ulster University, Bamford Centre
Keywords:	MENTAL HEALTH, MEDICAL EDUCATION & TRAINING, Burnout, Professional, GENERAL MEDICINE (see Internal Medicine)

SCHOLARONE™
Manuscripts

I, the Submitting Author has the right to grant and does grant on behalf of all authors of the Work (as defined in the below author licence), an exclusive licence and/or a non-exclusive licence for contributions from authors who are: i) UK Crown employees; ii) where BMJ has agreed a CC-BY licence shall apply, and/or iii) in accordance with the terms applicable for US Federal Government officers or employees acting as part of their official duties; on a worldwide, perpetual, irrevocable, royalty-free basis to BMJ Publishing Group Ltd ("BMJ") its licensees and where the relevant Journal is co-owned by BMJ to the co-owners of the Journal, to publish the Work in this journal and any other BMJ products and to exploit all rights, as set out in our licence.

The Submitting Author accepts and understands that any supply made under these terms is made by BMJ to the Submitting Author unless you are acting as an employee on behalf of your employer or a postgraduate student of an affiliated institution which is paying any applicable article publishing charge ("APC") for Open Access articles. Where the Submitting Author wishes to make the Work available on an Open Access basis (and intends to pay the relevant APC), the terms of reuse of such Open Access shall be governed by a Creative Commons licence – details of these licences and which Creative Commons licence will apply to this Work are set out in our licence referred to above.

Other than as permitted in any relevant BMJ Author's Self Archiving Policies, I confirm this Work has not been accepted for publication elsewhere, is not being considered for publication elsewhere and does not duplicate material already published. I confirm all authors consent to publication of this Work and authorise the granting of this licence.

For peer review only

Abstract

Objective: There is a dearth in suicide literature addressing the impact on general practitioners of losing a patient. We aimed to examine the personal and professional impact as well as the availability of support and why GPs did or did not use it.

Design: A qualitative study using one to one interviews with participants recruited using snowball sampling.

Setting: The study was conducted in a primary care setting.

Participants: Interviews were held with 19 GPs who have experienced the loss of two or more patients to suicide.

Results: GPs are impacted both personally and professionally when they lose a patient to suicide, but may not access formal help due to commonly-held idealised notions of a "good" GP who is regarded as having solid unperturbability. Fear of professional repercussions also plays a major role in deterring help-seeking.

Conclusions: There is a need for a systemic culture shift within general practice which allows doctors to seek support when their physical or mental health require it. This may help prevent stress, burnout and early retirement.

For peer review only

Strengths and limitations of this study

- The qualitative methodology employed in this study allows the depth of experience to be accessed in an area not widely researched in a population not comfortable discussing this topic.

- The study explored experiences of patient suicide within the context of primary care in NI, therefore these findings cannot be extrapolated to other medical settings and are not representative worldwide.
- For a more inclusive picture, further GPs, psychiatrists, psychologists and other suicide professionals should be incorporated in the research to offer additional insight and understanding.
- The emotional aspect of guilt and self-scrutiny following the death of a patient by suicide, may have contributed to potential under-reporting on the effects of patient suicide.

For peer review only

General Practitioners and patient suicide: A qualitative study investigating the professional and personal effects.

Introduction

It estimated up to 90% of people who complete suicide consult their GP shortly before death, with 20% - 76% seeing their GP in the month prior. [1] Impacts on GPs are both personal and professional. [2] Understanding these impacts may facilitate the implementation of support needed to allow GPs to understand and recover from patient suicide- induced trauma. There is a dearth in suicide literature addressing the reactions of GPs to this loss. [3-5]

A 2020 systematic review assessed the impact on mental health practitioners when they experienced this loss.[2] Guilt, blame, shock, anger, sadness and grief were common[2], in addition to lowered mood, poor sleep and increased irritability.[6] Professional reactions included doubt about practice and decision-making, [7] more caution in the management of suicide risk[8] and consideration of retirement or career change.[9] Similar research investigated the effects on frontline staff reporting that staff questioned themselves wondering if they could have helped more.[10]

Studies have examined the effects on psychiatrists and psychologists[11] with some focussing on GPs. In Pavlič et al.'s qualitative study of Slovenian GP experiences (n=22) GPs needed support after a patient suicide but reported barriers including lack of provision and personal hesitation due to perceptions of weakness and professional failure - leading GPs to only seek support from close colleagues.[4] Zambrano and Barton[12] reported that GP coping mechanisms included speaking with colleagues and acceptance of the death as unavoidable. A UK based study using 198 semi-structured interviews with GPs noted a lack of institutional understanding and poor professional support.[5]

For peer review only

Another UK based study focused on GPs experiences of dealing with parents bereaved by suicide while considering the impact on GPs[13]. They described personal grief at losing a patient they had cared for. The stigma of GPs accessing formal support and reliance on colleague support were consistent themes. A survey of 152 rural GPs in the Republic of Ireland[14] revealed GP reactions included feelings of guilt, sleep disruption, increased readiness to make psychiatric referrals in patient care and reduced confidence in decision making.

In summary, GPs experiences of suicide affect them on a personal level and inform how they care for future patients at risk of suicide and may influence them to leave general practice. This has implications on a personal, practice level and regional service provision level. This study adds to the literature addressing how GPs react to the suicide of patients by exploring the impact at a personal and professional level using interviews with nineteen GPs in Northern Ireland. It asked two main questions: What was the personal and professional impact of patient suicide on GPs? What was the availability of support and the reasons for access or avoidance?

Method

Design

The study 'Understanding Suicide and Help-Seeking in Urban and Rural Areas in Northern Ireland'[15] received ethical approval from the Office of Research Ethics Committees Northern Ireland (REC reference: 10/NIR03/65) and the main report provides full details of the methodology. This study employed qualitative data analysis of in-depth interviews (n=19) to gain an understanding of the impact of patient suicide on practising GPs, support that was available and support needed.

For peer review only

Participants

GPs who were working in NI and experienced the suicide of at least one patient in their care were invited to participate in the study via the Royal College of General Practitioners (NI). Nineteen GPs took part (n=11 women and n=8 men) from a range of primary care settings, (socio-economic and urban, semi-urban and rural populations) recruited via snowball sampling. Participants were long-serving practitioners (15-30 years' experience), involved in the care of more than 2 patients who died by suicide, several GPs indicated more than 10 cases, and three had personal experiences of suicide. The wider study explored specifically: (1) contact, recognition and management of suicidal patient; (2) primary care liaison with psychiatric and other services; and (3) recommendations for improved care and suicide prevention. The current analysis explored: (1) personal and professional impact of patient suicide on GPs; and (2) support available and support needed for GPs affected by suicide.[15]

Procedure and Data analysis

Qualitative data from interviews were analysed using inductive, thematic analysis[16] via NVivo12.[17] Data were analysed via a six-phase process: data familiarisation, generating initial codes, searching for themes, reviewing the themes, defining and naming the themes and report writing.[16] Three researchers (KN, GMA, AD) read transcripts twice for familiarisation, then independently generated initial codes and developed themes via repetition of text within the transcripts facilitating the development of main themes. Researchers independently analysed/coded the transcripts in batches of 5 and then met to review and develop broader themes. Codes were revised at 3 stages. All transcripts were re-analysed using the broader themes. Final analyses determined the association between themes and identified main themes and sub-themes.

For peer review only

Results

Analysis of the data identified five themes; rumination, personal reaction, professional impact, support that GPs accessed and barriers to accessing support. Themes and sub-themes are shown in Table 1.

[Table 1 here]

Most GPs had been in practice for more than 20 years with knowledge of multiple suicides, in practice and in their personal lives. GPs had experienced the loss of siblings, other family members, friends and colleagues. They supported family members of patients, close colleagues and other members of their practice. The impact of a suicide was variously described as '*devastating'* or '*very painful*'. No GP was unaffected by the suicide of a patient and effects were negative. Several GPs became distressed during interviews.

Five patient suicide typologies were interwoven into the experiences recounted: (1) Avoidable deaths: Regarded by GPs as people who might still be alive had services acted more swiftly or were better coordinated; (2) Missed signs: Suicides signalled by the patient but the GP recognised only in hindsight. (3) Valued patients: Suicide of patients who had coped with chronic illness with cheerful stoicism; (4) Apparent improvement: Suicides that occur despite apparent good mood and social functioning; (5) Family complaint: GP feels scapegoated by the family.

Rumination

All GPs reported a period of intense reflection after the loss during which they ruminated on the events preceding the suicide because "*there is always a huge guilt issue around suicide*." [GP7]. One GP sums up this dynamic and the need for reassurance.

For peer review only

> *"You want to know it's not your fault. You feel guilty because there's always something you could do… or maybe there isn't but you always feel that." [GP4]*

Different conclusions were reached seeking to quiet that voice that suggested that "*there's always something you can do."* [GP1]. These depended on assessment of prior events and relationships between the patient, GP, and other services. Where there had been a history of self-harm, alcohol or substance abuse, poor engagement with services or impulsive behaviour, GPs indicated that these deaths were upsetting but not shocking and they could not have changed the outcome; "*when you reflect on what could you have done differently that might have had an impact, most of them you couldn't*."[GP5] This recognition did not always permit feelings of failure to be resolved.

Other GPs concluded the outcome could have been changed. One GP working in an area of high deprivation reflected on the death of a young patient with a history of psychiatric problems who had been seen by several services in the days leading to their suicide. The patient stated they were suicidal and had shown signs of being suicidal during a home visit. Although services were alerted, the patient died by suicide. The GP felt that the system had failed.

> *"I was very upset about it because I felt that if he had been immediately sectioned he would have been alive..."[GP1]*

Others had the anxiety of never knowing if alternative action could have altered the outcome. One GP talks about patients who "*leave no footprints in the snow*" [GP19]. Another GP believes that,

> *"…most people who end up committing suicide, there probably is something documented … about their mood."[GP14]*

For peer review only

One GP described the experience of losing a young person who had previously presented with suicidal ideation. Although in treatment they showed no signs of being actively suicidal; they regularly attended the practice but consultations were about minor physical issues. The loss of the patient "*was not a predicted suicide"* [GP12] and it "*really shocked the whole practice."* [GP12]. Hindsight revealed signs that were not recognised and they were left feeling that "*in retrospect maybe things were more difficult."* [GP12] than the patient suggested. These questions remained unresolved, leaving feelings of guilt.

One difficultly expressed by GPs was the non-return of a patient whose mental state was concerning. GPs may assume that the problem is resolved, for patients the opposite may be true.

> *"I suppose as a GP you always assume that patients don't come back to see you if they're better, and that often is not the case, especially with mental health." [GP3]*

Part of the reason that GPs do not follow up is due to the nature and management of their workload. GPs describe an overloaded day with little room to spend time with patients and none for activities like following up patients they may have had a concern about; a 'niggle'. GPs do not know if they could have prevented a suicide but they also don't know the optimal criteria for intervention, how or when to intrude - "*you're never going to find out*" [GP4]. It can be very hard to know when the steps taken saved a life, but they will always know when they did not.

Personal Reaction

GPs reported overwhelmingly that the suicide of a patient "*has a massive impact on the clinician as a person*" [GP11]. The work of the average GP means that they will encounter what one described as 'endless human misery', with a GP describing the impact:

For peer review only

"Your whole life is absorbing. … you absorb everything … everybody's pain and sorrow …"[GP9]

Feelings expressed by GPs when they lost a patient included shock, loss, anger, upset, distress, desperation and sadness. The strongest feelings were responsibility, guilt and failure, "*there is an awful lot of guilt and everybody feels guilty*"[GP12]. Several GPs said they had experienced periods of depression and had witnessed colleagues being "*destroyed*" in the aftermath.

GPs expressed that they found the process of confirming the death to be "*very, very troubling*" [GP2] and in a manner resonating with post-traumatic stress disorder, described how it became "*a very visual memory*" [GP2] which remained with them. After this initial reaction came short term feelings. One GP talked about the suicide of an elderly patient who had started exhibiting erratic behaviour. The patient was engaged with services at the time with professionals disagreeing over a diagnosis. The GP felt the patient had fallen into "*the gaps of the system*" [GP11] The short-term impact on the GP was devastating.

"I came in and did my work and I went home and I cried, probably for about two weeks, all night." [GP11]

Long term impacts sometimes meant that even years after, the suicide was experienced as a painful memory. One GP described the "*intensely harrowing*" experiences of a close colleague who had experienced several "*traumatic suicides*", as a "*thinly covered scar*" [GP6] implying a wound that has never really healed. Another long-time impact is when other suicides occur "*it brings back the memories then of the others"* [GP7].

For peer review only

Professional Impact

There was a professional impact of a patient's suicide. For most these effects provided the catalyst to change the practice management of suicidality, but several described how patients' suicide had provoked early retirement of colleagues. We noted considerable differences in how the aftermath managed. Some practices had standard procedures such as conducting an event analysis to see "*whether we could have done anything differently or we could change our system*" [GP14] or meeting with other professionals involved for reviews. Even if there was no formal procedure, GPs performed some level of analysis. A GP who had experienced the death of their elderly patient described how they reviewed their notes with "*a fine-tooth comb*" [GP11]. These processes allowed GPs to explore and put in context the events leading up to the suicide.

In relation to risk management, one complication was knowing the full picture. Some GPs became aware patients were telling families a different version of events than in consultations. One GP described how a family member was given "*a very different picture"* [GP12] compared to the GP around the patient's state of mind and what help had been both offered and rejected by the patient. Another GP revealed that they may have "*a lot of people pretending to be mentally ill to get disability living allowance*" [GP9]. There are complicated personal and social factors that influence GPs judgement and decision-making.

A commonly reported impact was heightened vigilance.

> *"I would say I am much more vigilant and I'm now not the little kind of shy GP who is afraid to ask questions." [GP7]*

This extended to risk factors outside of the patient themselves such as a previous suicide in the family. It was not restricted to patients who presented with mental health issues, for example, it could be extended to patients in the same age range as the patient who had died.

For peer review only - http://bmjopen.bmj.com/site/about/guidelines.xhtml

"if I have a younger person in consultation now, I am looking at the mental health issues … prior history of any mental health issues" [GP12]

Commonly expressed and contributing to feelings of guilt and responsibility was 'blame culture' - GPs felt they were being held accountable for patients' ill-health and their 'failure' to cure people often resulted in blame. GPs found this demoralising and professionally devaluing.

"People with no medical education, people with too much medical education, but everyone loves bashing GPs" [GP4]

One GP who treated a young man shared the experience of being blamed when he died. The parents openly censured the GP, reproaching them for not doing more. The GP felt the parents had unrealistic expectations of primary care services and the extent of help available:

"… there was huge blame. The parents felt that we should have gone to their house and done more."[GP6]

Psychological Support

Given the intensity of GP's response, support is vital in assisting doctors to process the experiences and reducing negative impacts on future practice or premature retirement. Instead of a robust and easily accessible GP support system it appears that the health system makes scant allowance for the impact of a traumatic event on the GP; most participants suggest that they are obliged to '*just get on with it*'. There is a strong message that it is hard for GPs to seek professional help for this loss. GPs stated a lack of concern or compassion for them in the immediate aftermath, describing an expectation that they would be able to continue working with no reaction. An expectation they resented and found uncaring.

For peer review only

> *"The thing is we came back and just worked the rest of the shift… I just remember coming in the next day and no-one even saying, "that must have been tough."" [GP2]*

The most often used support was informal, found within GPs own homes or their own practice. No GP reported that they used professional support. Married GPs found support from their domestic partner. Others turned to close colleagues although this was often described in terms of a debriefing of a difficult case rather than a personal level of emotional support. GPs described how general practice can be "*reasonably isolated*." One reported that they felt the impact of a patient suicide was more damaging for them than for other mental health professionals such as psychiatry. The GP is seen as "*all things to all men*" [GP6], a person who can cure anything. There is the expectation in psychiatry that no matter how hard you try, you will lose patients in this way "*whereas in general practice we don't find it easy to accept failure.*" [GP6]. GPs are closer to the local community, have personal contacts and may be involved with care of other family members.

> *"you're not just involved with that person but you're involved with the whole family, and that's real general practice…" [GP7]*

Barriers to support

Three sub-themes were identified for this theme. Firstly, differing knowledge between GPs of available services. Some believed support by occupational health was available, others were sure it wasn't. One reason that GPs did not fully understand what help is available them professionally is because they would be highly unlikely to consider using it. Even when there was knowledge about support services available, GPs stated it would not be accessed.

> *"The BMA have a good counselling service by telephone … it never occurred to me to use it." [GP6]*

For peer review only

A second theme was that for GPs it is not acceptable to admit that sometimes those who help others, need to be helped themselves.

"GPs don't go sick. They work through ridiculous things because there's this macho "we're tough." "[GP6]

An over-valued work ethic or sense of duty was referred to as "*the hidden contract*" or "*the hidden curriculum*" - described as an understanding that "*I will not tell you if I feel bad and I will not ask you if you feel bad*". It is a cultural concept that is absorbed during training. It appears to be strongly associated to an internalised perception of the GP as a healer, a source of strength, but not someone who may need support and care. While at one level this appears to be a deeply held cultural trope, on a more direct level, GP mental health issues may impinge on the doctor's fitness to practice.

In the final sub-theme we noted concerns related to professional repercussions if a GP accessed help related to their own mental health and/or ability to cope. They worry that their fitness to practice (and their career) will be jeopardised.

"…if you had a sore knee, that wouldn't be an issue; but if you admit to depression there is a big question mark about your fitness to practise."[GP10]

The GPs overwhelmingly indicated they would not consider using support but they also said they knew it was needed;

"we need more support when suicides have happened… it needs to be built into the culture that we accept things when these things happen." [GP6]

To conclude, GPs undergo these complicated experiences when they lose patients to suicide. They spend time in intense personal and professional reflection. They may feel that a patient

For peer review only

was lost who did not need to be or conclude that they could not have helped. GPs are left with a profound and lasting sense of guilt, responsibility and failure. They may blame themselves for the death, they may be blamed by others. Mistakes may have been made. This is often channelled into taking action to try and ensure it does not happen again. Sometimes it does and the need for support increases. GPs know that they need support, but they don't feel they can access it. The age-old question remains, "who heals the healer?".

Discussion

Previous studies have examined the impact of patient suicide on psychiatric staff but fewer studies have examined specifically GPs reactions. We sought to explore personal and professional impacts, and support systems available for GPs who experienced patient suicide. We identified five themes: rumination, personal reaction, professional impact, psychological support and barriers to accessing support. The themes are similar to those uncovered in studies of suicide impact on other medical professionals. Experiencing a period of rumination or "*great self-scrutiny"* as described by Saini et al.[5] with similar personal and professional impacts. Guilt, blame, shock, anger, sadness and grief following the suicide of a patient are commonly reported.[2] GP distress was noted by Foggin et al.[13] and Saini et al.[5] The current study additionally indicates the considerable psychological and organisational barriers to GP support following patient suicide. We noted a significant dissonance between the GP self-image of solid unperturbability, and the distress caused by patient suicide. The idealised estimation of a 'good' GP embodied in a *'hidden contract*' develops in training and is reinforced over years but in practice but appears to fail in the long-term with sustained and intimate contact with patients. A level of objectivity or detachment from the patient is a form of self-protection that may appear sensible and attractive in training but deteriorates over time. One GP contrasted the loss of a patient to suicide in general practice to that experienced in psychiatry where there is a commonly held expectation that some patients will

For peer review only

die by suicide. Regular contact with high-risk patients increases this expectation. In primary care where suicide is relatively rare a patient's suicide is more likely to undermine the professional identity of GPs who work as individuals rather than part of a clinical team.

The "*hidden contract*" discourages GP help-seeking for psychological support. It is seen as a weakness. This fear of being diminished by loss of regard by others has been described in other studies of self-care and intra-professional support among GPs and other high visibility caring professions in which coping, self-reliance and resilience may be over-valued to the detriment of the individual.[18 19]

GPs describe a high-pressure environment with an excessive workload all swiftly managed in consecutive ten-minute appointments. In this context it is difficult to provide compassionate care which requires time to listen in order to understand. GPs are conflicted by systemic requirements for example they might wish to request hospital admission for people whose mental health has deteriorated knowing this is in opposition to the policy of community care. Other role conflicts are the amount of time spent with individuals should be dictated by patients' needs but can't be due to time demands and pressures. GPs may want to acknowledge the distress of bereaved patients but seek to convey a detached professionalism.

Cheshire et al.[20] used a qualitative design to understand how GPs coped with workplace challenges and stresses. Among these were the inability to connect authentically due to time demands and requirements such as the need to record patient indicators within the ten minutes appointment. GPs felt primary care was functioning in more detached ways which are more comparable with secondary care functioning. Some GPs felt that working longer hours was the most effective way they could deal with the sense of being overwhelmed, to the detriment of a work-life balance. Strategies which the GPs used to mitigate work stress included meditation, mindfulness, stress management techniques, taking regular exercise,

For peer review only

eating well and meeting and debriefing with their colleagues however the study also reported that a surprisingly high number of participants had adopted changes such as reducing their working hours or changing their role to locum to allow them to downsize and regain work-life balance.[20]

Emotional hardening and cynicism among GP appears ubiquitous. One GP described going to identify a body after a suicide and on their return to a busy day and a list of patients, "*no-one even asked*" how they were, suggesting a resigned fatalism among the profession. Perhaps exacerbating the self-neglect GPs were concerned about the professional repercussions that might accompany accessing support. GPs raised the issue of potentially being declared unfit to practise if formal help was sought, a finding supported by previous research. Spiers et al.[21] found that GPs reported issues around privacy, confidentiality and the stigma surrounding seeking help. This worry around professional repercussions begins with medical training and is reported by medical students.[22]

The lack of uptake of services for mental health issues in the wake of the loss resonates with low GP uptake of care when physically ill in a study reported by Thompson and colleagues.[19] GPs want help but don't feel they can access it. The impact of this inability to seek help is seen in studies conducted on the mental health of doctors. Doctor's experience high rates of anxiety,[23] depression,[24] substance misuse,[25]emotional exhaustion and burnout[26] and are at high risk of suicide themselves.[22] Male doctors are 1-4 times more likely than other men to die by suicide and female doctors 2-3 times more likely than other women.[27]

GP uncertainty about the availability of support may indicate their own ambivalence about help-seeking or the inappropriateness of current available support. While GPs are seen as part of the National Health Service in the UK, they are individual practitioners who work as self-employed practitioners within GP surgeries. One review of the literature recommended that

For peer review only

doctors require specialist services which focus on the unique and complex situation of a doctor becoming a patient.[28] George et al.[29] outline a pilot aimed at preventing or minimising mental health issues in doctors. This strategy would operate at a primary, secondary and tertiary level. Primary level prevention would be aimed mainly at medical students and may work to counteract the macho culture in which a doctor is expected to show no weakness. This level would include awareness-raising, enhancing coping skills and stress management skills. Secondary level prevention would allow early diagnosis and intervention and would include measures for screening, reducing stigma and creating a supportive culture for doctors. The tertiary level would include offering treatments and interventions to minimise the chronic negative effects of mental ill health on doctors.

Conclusions

The findings of this study suggest a need to provide GPs with the appropriate psychological support after losing a patient to suicide. Despite a wish to remain true to the GP self-image of solid unperturbability created by adherence to the 'hidden contract', GPs report that the loss of a patient to suicide results in a period of self-scrutiny. From this may come short or longer term effects on their mental health. The "*hidden contract*" translates into doctors being unable to comfortably make a transition to patient which means that doctors are unable to properly receive the care they seek to provide for others.

Creating a culture shift which allows doctors to complete this transition will help prevent burnout and early retirement from a system which is not currently supporting them. The price for seeking that help should not include that their fitness to practice will be questioned consequently. GPs work in a high risk, high stress environment in which they are likely to experience loss which will require intervention and support. We echo the call to provide a strategy which will facilitate the culture change needed and implement the services needed to

For peer review only

support our doctors in general practice, including guidelines for GPs on when and how to ask for help. If a change is not facilitated, we will continue to see GPs experiencing distress and mental health issues which when unaddressed are contributing to mental illness, burnout and early retirement.

Contributors GMcA, KN and GL were responsible for data analysis and interpretations. GMcA wrote the initial draft of the article. GMcA revised the article in response to the comments of the co-authors after they commented critically on the draft. All authors gave their final approval of the version to be published.

Funding This study was funded by the Public Health Agency in Northern Ireland

Competing Interests None declared.

For peer review only

References

1. Leavey, G., Rosato, M., Galway, K., Hughes, L., Mallon, S., & Rondon, J. (2016). Patterns and predictors of help-seeking contacts with health services and general practitioner detection of suicidality prior to suicide: a cohort analysis of suicides occurring over a two-year period. *BMC psychiatry*, *16*, 1-8.

2. Sandford, D. M., Kirtley, O. J., Thwaites, R., & O'Connor, R. C. (2020). The impact on mental health practitioners of the death of a patient by suicide: a systematic review. In *Clinical Psychology & Psychotherapy* (Vol. 2). https://doi.org/10.1002/cpp.2515

3. Leavey, G., Mallon, S., Rondon-Sulbaran, J., Galway, K., Rosato, M., & Hughes, L. (2017). The failure of suicide prevention in primary care: family and GP perspectives–a qualitative study. *BMC psychiatry*, *17*(1), 1-10.

4. Pavlič, D. R., Treven, M., Maksuti, A., Švab, I., & Grad, O. (2018). General practitioners' needs for support after the suicide of patient: A qualitative study. *European Journal of General Practice*, *24*(1), 177–182. https://doi.org/10.1080/13814788.2018.1485648

5. Saini, P., Chantler, K., While, D., & Kapur, N. (2016). Do GPs want or need formal support following a patient suicide?: A mixed methods study. *Family Practice*, *33*(4), 414–420. https://doi.org/10.1093/fampra/cmw040

6. Alexander, D. A., Klein, S., Gray, N. M., Dewar, I. G., & Eagles, J. M. (2000). Suicide by patients: Questionnaire study of its effect on consultant psychiatrists. *British Medical Journal*, *320*(7249), 1571–1574. https://doi.org/10.1136/bmj.320.7249.1571

7. Murphy, P. T., Clogher, L., Van Laar, A., O'Regan, R., McManus, S., McIntyre, A., O'Connell, A., Geraghty, M., Henry, G., & Hallahan, B. (2019). The impact of service

For peer review only

user's suicide on mental health professionals. *Irish Journal of Psychological Medicine, April 2020*. https://doi.org/10.1017/ipm.2019.4

8. Finlayson, M., & Graetz Simmonds, J. (2018). Impact of Client Suicide on Psychologists in Australia. *Australian Psychologist*, *53*(1), 23–32. https://doi.org/10.1111/ap.12240

9. Gibbons, R., Brand, F., Carbonnier, A., Croft, A., Lascelles, K., Wolfart, G., & Hawton, K. (2019). Effects of patient suicide on psychiatrists: survey of experiences and support required. *BJPsych Bulletin*, *43*(5), 236–241. https://doi.org/10.1192/bjb.2019.26

10. Gaffney, P., Russell, V., Collins, K., Bergin, A., Halligan, P., Carey, C., & Coyle, S. (2009). Impact of patient suicide on front-line staff in Ireland. *Death Studies*, *33*, 639-656.

11. Foley, S. R., & Kelly, B. D. (2007). When a patient dies by suicide: Incidence, implications and coping strategies. *Advances in Psychiatric Treatment*, *13*(2), 134–138. https://doi.org/10.1192/apt.bp.106.002501

12. Zambrano, S. C., & Barton, C. A. (2011). On the journey with the dying: how general practitioners experience the death of their patients. *Death Studies*, *35*(9), 824–851.

13. Foggin, E., McDonnell, S., Cordingley, L., Kapur, N., Shaw, J., & Chew-Graham, C. A. (2016). GPs' experiences of dealing with parents bereaved by suicide: A qualitative study. *British Journal of General Practice*, *66*(651), e737–e746. https://doi.org/10.3399/bjgp16X686605

14. Halligan, P., & Corcoran, P. (2001). The impact of patient suicide on rural general practitioners. *British Journal of General Practice*, *51*(465), 295–296.

15. Leavey, G., Galway, K., Hughes, L., Mallon, S., Sulbarán, J. R., & Rosato, M. (2016). Understanding Suicide and Help-Seeking in Urban and Rural Areas in Northern

For peer review only

Ireland. *Bamford Centre for Mental Health and Wellbeing, Ulster University: Belfast, UK.*

16. Braun, V., & Clarke, V. (2006). Using thematic analysis in psychology. *Qualitative research in psychology*, *3*, 77-101.

17. Hoover R. S., & Koerber, A. L. (2011). Using NVivo to answer the challenges of qualitative research in professional communication: Benefits and best practices tutorial. Transactions on Professional Communication, 54, 68–82.

18. Leavey, G. (2008). UK clergy and people in mental distress: Community and patterns of pastoral care. *Transcultural Psychiatry*, *45*, 79-104.

19. Thompson, W. T., Cupples, M. E., Sibbett, C. H., Skan, D. I., & Bradley, T. (2001). Challenge of culture, conscience, and contract to general practitioners' care of their own health: qualitative study. *BMJ: British Medical Journal*, *323*(7315), 728.

20. Cheshire, A., Ridge, D., Hughes, J., Peters, D., Panagioti, M., Simon, C., & Lewith, G. (2017). Influences on GP coping and resilience: a qualitative study in primary care. *British Journal of General Practice*, *67*, e428-e436.

21. Spiers, J., Buszewicz, M., Chew-Graham, C. A., Gerada, C., Kessler, D., Leggett, N., ... & Riley, R. (2017). Barriers, facilitators, and survival strategies for GPs seeking treatment for distress: a qualitative study. *British Journal of General Practice*, *67*, e700-e708.

22. Devi, S. (2011). Doctors in distress. *The Lancet*, *377*(9764), 454-455.

23. Kroenke, K., Spitzer, R. L., Williams, J. B., Monahan, P. O., & Löwe, B. (2007). Anxiety disorders in primary care: prevalence, impairment, comorbidity, and detection. *Annals of internal medicine*, *146*, 317-325.

For peer review only

24. Firth-Cozens, J. (2001). Interventions to improve physicians’ well-being and patient care. *Social science & medicine*, *52*, 215-222.

25. Bennett, J., & O'Donovan, D. (2001). Substance misuse by doctors, nurses and other healthcare workers. *Current opinion in Psychiatry*, *14*, 195-199.

26. Rø, K. E. I., Gude, T., Tyssen, R., & Aasland, O. G. (2008). Counselling for burnout in Norwegian doctors: one year cohort study. *BMJ*, *337*.

27. Schernhammer, E. S., & Colditz, G. A. (2004). Suicide rates among physicians: a quantitative and gender assessment (meta-analysis). *American Journal of Psychiatry*, *161*, 2295-2302.

28. Brooks, S. K., Gerada, C., & Chalder, T. (2011). Review of literature on the mental health of doctors: are specialist services needed?. *Journal of Mental Health*, *20*, 146-156.

29. George, S., Deshpande, S., & Kallivayalil, R. A. (2020). Are specialist treatment services needed for doctors with mental health problems?.*Indian Journal of Psychological medicine*, *42*, 105-107.

For peer review only

For peer review only

Table 1: Summary of Themes and Sub-Themes Generated from GP Interviews

Themes	Sub-themes
1. Rumination	They could not have changed the outcome.<br>They could have changed the outcome.<br>The feeling of never knowing.
2. Personal reaction	Viewing a body.<br>Short term feelings.<br>Long term feelings.
3. Professional impact	Analysis of events<br>Improvements to practice.<br>Blame culture.
4. Psychological support	Family and friends .<br>Within the practice.<br>Other services.
5. Barriers to accessing support	Knowledge of services.<br>The hidden contract.<br>Professional repercussions.

For peer review only

BMJ Open

A qualitative study investigating the professional and personal effects of patient suicide on General Practitioners in Northern Ireland.

Journal:	*BMJ Open*
Manuscript ID	bmjopen-2023-077940.R1
Article Type:	Original research
Date Submitted by the Author:	08-Jan-2024
Complete List of Authors:	McAnee, Grainne; Ulster University, School of Psychology<br>Norwood, Kelly; Ulster University Faculty of Life and Health Sciences, School of Psychology<br>Leavey, Gerard; University of Ulster, Psychology
**Primary Subject Heading**:	General practice / Family practice
Secondary Subject Heading:	Qualitative research, Mental health
Keywords:	MENTAL HEALTH, MEDICAL EDUCATION & TRAINING, Burnout, Professional, GENERAL MEDICINE (see Internal Medicine)

SCHOLARONE™
Manuscripts

I, the Submitting Author has the right to grant and does grant on behalf of all authors of the Work (as defined in the below author licence), an exclusive licence and/or a non-exclusive licence for contributions from authors who are: i) UK Crown employees; ii) where BMJ has agreed a CC-BY licence shall apply, and/or iii) in accordance with the terms applicable for US Federal Government officers or employees acting as part of their official duties; on a worldwide, perpetual, irrevocable, royalty-free basis to BMJ Publishing Group Ltd ("BMJ") its licensees and where the relevant Journal is co-owned by BMJ to the co-owners of the Journal, to publish the Work in this journal and any other BMJ products and to exploit all rights, as set out in our licence.

The Submitting Author accepts and understands that any supply made under these terms is made by BMJ to the Submitting Author unless you are acting as an employee on behalf of your employer or a postgraduate student of an affiliated institution which is paying any applicable article publishing charge ("APC") for Open Access articles. Where the Submitting Author wishes to make the Work available on an Open Access basis (and intends to pay the relevant APC), the terms of reuse of such Open Access shall be governed by a Creative Commons licence – details of these licences and which Creative Commons licence will apply to this Work are set out in our licence referred to above.

Other than as permitted in any relevant BMJ Author's Self Archiving Policies, I confirm this Work has not been accepted for publication elsewhere, is not being considered for publication elsewhere and does not duplicate material already published. I confirm all authors consent to publication of this Work and authorise the granting of this licence.

For peer review only

Abstract

Objective: There is a dearth in suicide literature addressing the impact on general practitioners of losing a patient. We aimed to examine the personal and professional impact as well as the availability of support and why GPs did or did not use it.

Design: A qualitative study using one to one interviews with participants recruited using snowball sampling.

Setting: The study was conducted in a primary care setting.

Participants: Interviews were held with 19 GPs within primary care in Northern Ireland.

Results: GPs are impacted both personally and professionally when they lose a patient to suicide, but may not access formal help due to commonly-held idealised notions of a "good" GP who is regarded as having solid unperturbability. Fear of professional repercussions also plays a major role in deterring help-seeking.

Conclusions: There is a need for a systemic culture shift within general practice which allows doctors to seek support when their physical or mental health require it. This may help prevent stress, burnout and early retirement.

Strengths and limitations of this study

- The qualitative methodology employed in this study allows the depth of experience to be accessed in an area not widely researched in a population not comfortable discussing this topic.

For peer review only

- The study explored experiences of patient suicide within the context of primary care in NI, therefore these findings cannot be extrapolated to other medical settings and are not representative worldwide.
- For a more inclusive picture, further GPs, psychiatrists, psychologists, nurses, community pharmacists and other professionals should be incorporated in the research to offer additional insight and understanding.
- The emotional aspect of guilt and self-scrutiny following the death of a patient by suicide, may have contributed to potential under-reporting on the effects of patient suicide.

For peer review only

A qualitative study investigating the professional and personal effects of patient suicide on General Practitioners in Northern Ireland.

Introduction

It estimated up to 90% of people who die by suicide consult their GP shortly before death, with 20% - 76% seeing their GP in the month prior.(1) Impacts on GPs are both personal and professional.(2) Understanding these impacts may facilitate the implementation of support needed to allow GPs to understand and recover from patient suicide- induced trauma. There is a dearth in suicide literature addressing the reactions of GPs to this loss.(3-5)

A 2020 systematic review assessed the impact on mental health practitioners when they experienced this loss.(2) Guilt, blame, shock, anger, sadness and grief were common(2), in addition to lowered mood, poor sleep and increased irritability.(6) Professional reactions included doubt about practice and decision-making,(7) more caution in the management of suicide risk(8) and consideration of retirement or career change.(9) Similar research investigated the effects on frontline staff reporting that staff questioned themselves wondering if they could have helped more.(10)

Studies have examined the effects on psychiatrists and psychologists(11) with some focussing on GPs. In Pavlič et al.'s qualitative study of Slovenian GP experiences (n=22) GPs needed emotional support after a patient suicide but reported barriers including lack of provision and personal hesitation due to perceptions of weakness and professional failure - leading GPs to only seek support from close colleagues.(4) Zambrano and Barton(12) reported that GP coping mechanisms included speaking with colleagues and acceptance of the death as unavoidable. A UK based study using 198 semi-structured interviews with GPs noted a lack of institutional understanding and poor professional support.(5)

For peer review only - http://bmjopen.bmj.com/site/about/guidelines.xhtml

Another UK based study focused on GPs experiences of dealing with parents bereaved by suicide while considering the impact on GPs.(13) They described personal grief at losing a patient for whom they cared. The stigma of GPs accessing formal support and reliance on colleague support were consistent themes. A survey of 152 rural GPs in the Republic of Ireland(14) who lost patients who died by suicide revealed GP reactions included feelings of guilt, sleep disruption, increased readiness to make psychiatric referrals in patient care and reduced confidence in decision making.

In summary, GPs experiences of suicide affect them on a personal level and inform how they care for future patients at risk of suicide. This study adds to the literature addressing how GPs react to the suicide of patients by exploring the impact at a personal and professional level using interviews with nineteen GPs in Northern Ireland. To the best of the author's knowledge this is the first qualitative study of this scale in Northern Ireland. It is one which explored access to support in this population in depth. Finally, it allows the needs of GPs in Northern Ireland to be better responded to. It asked two main questions: What was the personal and professional impact of patient suicide on GPs? What was the availability of support and the reasons for access or avoidance?

For peer review only

Method

Design

The study 'Understanding Suicide and Help-Seeking in Urban and Rural Areas in Northern Ireland'(15) received ethical approval from the Office of Research Ethics Committees Northern Ireland (REC reference: 10/NIR03/65) and the main report provides full details of the methodology. This qualitative study employed in-depth interviews (n=19) which were

For peer review only - http://bmjopen.bmj.com/site/about/guidelines.xhtml

recorded and transcribed to gain an understanding of the impact of patient suicide on practising GPs, support that was available and support needed. Informed consent was given after comprehensive information on the topic was shared with potential interviewees. Experienced researchers were employed with interpersonal skills suited to the topic, should interviewees become distressed during interviews they were reminded they were allowed to stop the interview at any time should they find it too upsetting.

Patient and public involvement

The project was supported by a Patient and Public Involvement (PPI) committee from it's conception through to conclusion. The committee advised on all stages of the project. This included formulation of the research questions, design, recruitment and how the findings would be disseminated.

Participants

The full study within which this is placed[15] interviewed both family members of people in Northern Ireland who died by suicide and GPs. GPs who were working in NI and experienced the suicide of at least one patient in their care were invited to participate in the research by letter, with a follow-up phone call, according to the geographical location of their practice in order to represent any disparities between urban and rural areas. After a number of unsuccessful attempts to recruit GPs through traditional postal invitations, the 19 GPs who participated in the study were recruited through a snowball sampling approach with support from the Royal College of General Practitioners (NI). The study explored: (1) contact, recognition and management of suicidal patient; (2) primary care liaison with psychiatric and other services; and (3) recommendations for improved care and suicide prevention

For peer review only

Procedure and Data analysis

Qualitative data from interviews were analysed using inductive, thematic analysis via NVivo12. Data were analysed via a six-phase process: data familiarisation, generating initial codes, searching for themes, reviewing the themes, defining and naming the themes and report writing. (16) Three researchers (KN, GMA, AD) read transcripts twice for familiarisation, then independently generated initial codes. Researchers independently coded transcripts in batches of 5 and then met to review and collapse codes. All transcripts were re-analysed using the final set of agreed codes from the iterative process. From these themes and sub-themes were generated and reviewed until consensus was reached.

Reflexivity

Qualitative researchers are required to make sense of the material they engage with(17) and as such it is imperative that they acknowledge how their own experiences and beliefs can impact their perspective. With such an emotive and delicate topic it was important to be mindful and take appropriate steps against this. Participants provided data on experiences which are powerful and important as well as deeply personal and the respect it was treated with included that it was approached with sensitivity and objectivity. It was important that researchers remain open to the possibility of findings being contradictory to personal feelings and beliefs(18). Qualitative research can involve the practice of analysts disregarding prior knowledge and experience(19) however this may not always be possible or indeed necessary[17]. The researchers adopted a practice of self-reflection and consensus of three researchers to protect robustness of findings. The use of a literature review before analysis and lacing findings within the context of that literature also protected the integrity of analysis.

Trustworthiness and credibility

For peer review only

For peer review only - http://bmjopen.bmj.com/site/about/guidelines.xhtml

Due to lack of standardisation of the methodologies employed in qualitative research it is not possible to conduct formal reliability testing however Savin-Baden and Major(20) provide criteria to improve trustworthiness which include methodological coherence (congruence between research objectives, methodologies, data and analysis), audit trail (description of the research process from beginning to end which can uncover other possible influences on findings), and negative case analysis (actively identifying and extracting data which was not compatible with the main or expected findings in order to encourage critical thinking and subsequent modification). The use of the Standards for Reporting Qualitative Research (SRQR) was adopted and provide to further the aim of trustworthiness.(21)

Results

Nineteen GPs took part (n=11 women and n=8 men) from a range of primary care settings, (socio-economic and urban, semi-urban and rural populations). Participants were long-serving practitioners (15-30 years' experience), involved in the care of more than 2 patients who died by suicide, several GPs indicated more than 10 cases, and three had personal experiences of suicide.

Analysis of the data identified four themes; personal reaction, professional impact, barriers to accessing support and psychological support. Themes and sub-themes are shown in Table 1, Figure 1 shows the relationships between them.

For peer review only - http://bmjopen.bmj.com/site/about/guidelines.xhtml

Table 1: Summary of Themes and Sub-Themes Generated from GP Interviews

Themes	Sub-themes
1. Personal reaction	Viewing a body.
	Short term feelings.
	Long term feelings.
2. Professional impact	Analysis of events
	Improvements to practice.
	Blame culture.
3. Barriers to accessing support	Knowledge of services.
	The hidden contract.
	Professional repercussions.
4. Psychological support	Family and friends .
	Within the practice.
	Other services.

For peer review only

[Figure 1 here]

Most GPs had been in practice for more than 20 years with knowledge of multiple suicides, in practice and in their personal lives. GPs had experienced the loss of siblings, other family members, friends and colleagues. They supported family members of patients, close

For peer review only - http://bmjopen.bmj.com/site/about/guidelines.xhtml

colleagues and other members of their practice. The impact of a suicide was variously described as '*devastating'* or '*very painful*'. No GP was unaffected by the suicide of a patient and effects were negative. Several GPs became distressed during interviews.

Five patient suicide typologies were interwoven into the experiences recounted: (1) Avoidable deaths: Regarded by GPs as people who might still be alive had services acted more swiftly or were better coordinated; (2) Missed signs: Suicides signalled by the patient but the GP recognised only in hindsight. (3) Valued patients: Suicide of patients who had coped with chronic illness with cheerful stoicism; (4) Apparent improvement: Suicides that occur despite apparent good mood and social functioning; (5) Family complaint: GP feels scapegoated by the family.

Personal Reaction

Most GPs reported that the suicide of a patient "*has a massive impact on the clinician as a person*" [GP11]. The work of the average GP means that they will encounter what one described as '*endless human misery*', with a GP describing the impact:

> *"Your whole life is absorbing. … you absorb everything … everybody's pain and sorrow …"[GP9]*

Feelings expressed by GPs when they lost a patient included shock, loss, anger, upset, distress, desperation and sadness. The strongest feelings were responsibility, guilt and failure, "*there is an awful lot of guilt and everybody feels guilty*"[GP12]. Several GPs said they had experienced periods of depression and had witnessed colleagues being "*destroyed*" in the aftermath.

For peer review only - http://bmjopen.bmj.com/site/about/guidelines.xhtml

GPs expressed that they found the process of confirming the death to be "*very, very troubling*" [GP2] and in a manner resonating with post-traumatic stress disorder, described how it became "*a very visual memory*" [GP2] which remained with them. After this initial reaction came short term feelings. One GP talked about the suicide of an elderly patient who had started exhibiting erratic behaviour. The patient was engaged with services at the time with professionals disagreeing over a diagnosis. The GP felt the patient had fallen into "*the gaps of the system*" [GP11] The short-term impact on the GP was devastating.

> *"I came in and did my work and I went home and I cried, probably for about two weeks, all night." [GP11]*

Short terms feelings were led by a period of intense reflection after the loss during which they ruminated on the events preceding the suicide because "*there is always a huge guilt issue around suicide*." [GP7]. One GP sums up this dynamic and the need for reassurance.

> *"You want to know it's not your fault. You feel guilty because there's always something you could do… or maybe there isn't but you always feel that." [GP4]*

Different conclusions were reached seeking to quiet that voice that suggested that "*there's always something you can do."* [GP1]. These depended on assessment of prior events and relationships between the patient, GP, and other services. Where there had been a history of self-harm, alcohol or substance abuse, poor engagement with services or impulsive behaviour, GPs indicated that these deaths were upsetting but not shocking and they could not have changed the outcome; "*when you reflect on what could you have done differently that might have had an impact, most of them you couldn't*."[GP5] This recognition did not always permit feelings of failure to be resolved.

For peer review only

Other GPs concluded the outcome could have been changed. One GP working in an area of high deprivation reflected on the death of a young patient with a history of psychiatric problems who had been seen by several services in the days leading to their suicide. The patient stated they were suicidal and had shown signs of being suicidal during a home visit. Although services were alerted, the patient died by suicide. The GP felt that the system had failed.

> *"I was very upset about it because I felt that if he had been immediately sectioned he would have been alive..."[GP1]*

Others had the anxiety of never knowing if alternative action could have altered the outcome. One GP talks about patients who "*leave no footprints in the snow*" [GP19]. This GP is describing patients who show no signs of considering suicide and leads the GP to wonder both,

> *"is it a switch that can flick in your head? And the natural follow-up from that is could that flick in anybody's head…"[GP19]*

The description used is suggestive of something very haunting with a sense of silence wrapped around it. Another GP believed that,

> *"…most people who end up committing suicide, there probably is something documented … about their mood."[GP14]*

One GP described the experience of losing a young person who had previously presented with suicidal ideation. Although in treatment they showed no signs of being actively suicidal; they regularly attended the practice but consultations were about minor physical issues. The loss of the patient "*was not a predicted suicide*" [GP12] and it "*really shocked the whole*

For peer review only

For peer review only - http://bmjopen.bmj.com/site/about/guidelines.xhtml

practice." [GP12]. Hindsight revealed signs that were not recognised and they were left feeling that "*in retrospect maybe things were more difficult."* [GP12] than the patient suggested. These questions remained unresolved, leaving feelings of guilt.

One difficultly expressed by GPs was the non-return of a patient whose mental state was concerning. GPs may assume that the problem is resolved, for patients the opposite may be true.

> *"I suppose as a GP you always assume that patients don't come back to see you if they're better, and that often is not the case, especially with mental health." [GP3]*

Part of the reason that GPs do not follow up is due to the nature and management of their workload. GPs describe an overloaded day with little room to spend time with patients and none for activities like following up patients they may have had a concern about; a 'niggle'. GPs do not know if they could have prevented a suicide but they also don't know the optimal criteria for intervention, how or when to intrude - "*you're never going to find out*" [GP4]. It can be very hard to know when the steps taken saved a life, but they will always know when they did not.

Long term feelings sometimes meant that even years after, the suicide was experienced as a painful memory. One GP described the "*intensely harrowing*" experiences of a close colleague who had experienced several "*traumatic suicides*", as a "*thinly covered scar*" [GP6] implying a wound that has never really healed. Another long-time impact is when other suicides occur "*it brings back the memories then of the others"* [GP7].

Professional Impact

For peer review only - http://bmjopen.bmj.com/site/about/guidelines.xhtml

There was a professional impact of a patient suicide. For most these effects provided the catalyst to change the practice management of suicidality, but several described how patients' suicide had provoked early retirement of colleagues. We noted considerable differences in how the aftermath was managed. Some practices had standard procedures such as conducting an event analysis to see "*whether we could have done anything differently or we could change our system*" [GP14] or meeting with other professionals involved for reviews. Even if there was no formal procedure, GPs performed some level of analysis. A GP who had experienced the death of their elderly patient described how they reviewed their notes with "*a fine-tooth comb*" [GP11]. These processes allowed GPs to explore and put in context the events leading up to the suicide.

There are complicated personal and social factors that influence GPs judgement and decision-making. In relation to risk management, one such factor was knowing the full picture. Some GPs became aware patients were telling families a different version of events than in consultations. One GP described how a family member was given "*a very different picture*" [GP12] compared to the GP around the patient's state of mind and what help had been both offered and rejected by the patient. Another GP revealed that they may have "*a lot of people pretending to be mentally ill to get disability living allowance*" [GP9].

A commonly reported impact was heightened vigilance.

> *"I would say I am much more vigilant and I'm now not the little kind of shy GP who is afraid to ask questions." [GP7]*

This extended to risk factors outside of the patient themselves such as a previous suicide in the family. It was not restricted to patients who presented with mental health issues, for example, it could be extended to patients in the same age range as the patient who had died.

For peer review only

"if I have a younger person in consultation now, I am looking at the mental health issues … prior history of any mental health issues" [GP12]

Commonly expressed and contributing to feelings of guilt and responsibility was 'blame culture' - GPs felt they were being held accountable for patients' ill-health and their 'failure' to cure people often resulted in blame. GPs found this demoralising and professionally devaluing.

"People with no medical education, people with too much medical education, but everyone loves bashing GPs" [GP4]

One GP who treated a young man shared the experience of being blamed when he died. The parents openly censured the GP, reproaching them for not doing more. The GP felt the parents had unrealistic expectations of primary care services and the extent of help available:

"… there was huge blame. The parents felt that we should have gone to their house and done more."[GP6]

Barriers to accessing support

Given the impact described above it was worrying that GPs reported concerns and described barriers to accessing formal support. Three sub-themes were identified. Firstly, differing knowledge between GPs of available services. Some believed support by occupational health was available, others were sure it wasn't. One reason that GPs did not fully understand what help is available them professionally is because they would be highly unlikely to consider using it. Even when there was knowledge about support services available, GPs stated it would not be accessed.

For peer review only

For peer review only - http://bmjopen.bmj.com/site/about/guidelines.xhtml

“The BMA have a good counselling service by telephone … it never occurred to me to use it.”[GP6]

A second sub-theme was that for GPs it is not acceptable to admit that sometimes those who help others, need to be helped themselves.

“GPs don't go sick. They work through ridiculous things because there’s this macho “we’re tough.” “[GP6]

An over-valued work ethic or sense of duty was referred to as “*the hidden contract*” or “*the hidden curriculum*” - described as an understanding that “*I will not tell you if I feel bad and I will not ask you if you feel bad*”. It is a cultural concept that is absorbed during training. It appears to be strongly associated to an internalised perception of the GP as a healer, a source of strength, but not someone who may need support and care. While at one level this appears to be a deeply held cultural trope, on a more direct level, GP mental health issues may impinge on the doctor’s fitness to practice.

In the final sub-theme we noted concerns related to professional repercussions if a GP accessed help related to their own mental health and/or ability to cope. They worry that their fitness to practice (and their career) will be jeopardised.

“…if you had a sore knee, that wouldn’t be an issue; but if you admit to depression there is a big question mark about your fitness to practise.“[GP10]

The GPs overwhelmingly indicated they would not consider using support but they also said they knew it was needed;

For peer review only

For peer review only - http://bmjopen.bmj.com/site/about/guidelines.xhtml

"we need more support when suicides have happened… it needs to be built into the culture that we accept things when these things happen." [GP6]

Psychological Support

Given the intensity of GP's responses to losing a patient to suicide and the barriers reported above the next question becomes centred on what support was available in assisting doctors to process the experiences and reducing negative impacts on future practice or potential premature retirement. If a GP does decide they would like support instead of a robust and easily accessible GP support system it appears that the health system makes scant allowance for the impact of a traumatic event on the GP; most participants suggested that they are obliged to just get on with it.

"…but pull up your socks and get on with it. This is your job; get on with it. You have to deal with this."[GP11]

There was a strong message that it is hard for GPs to seek professional help for this loss and a sense of conflict around it.

"I'm not saying GPs don't need it, I'm saying that GPs don't access it, and the reason I think they don't access it is twofold. I suppose GPs feel that I'm big enough… I'm strong enough, I don't need counselling. And second of all, there's an embarrassment issue of having to seek help yourself as someone who gives help to others. I think there is a difficulty there." [GP1

For peer review only

GPs stated a lack of concern or compassion for them in the immediate aftermath, one describing an expectation that they would be able to continue working with no reaction to what they describe as a traumatic event,

“there's a huge difference in terms hearing that someone has taken their own life and then being the GP who’s actually going out to confirm that person has passed away, and I think it’s one of the things, that it is fairly traumatic to see the person in that situation.”[GP2]

They found this expectation uncaring and resented it,

“The thing is we came back and just worked the rest of the shift… I just remember coming in the next day and no-one even saying, “that must have been tough.””[GP2]

The most often used support was informal. Some GPs found support within their own homes, for example married GPs found support from their domestic partner, but for most GPs they found support within their own practice.

“I suppose in my practice we have three GPs. We are all much of the same age group and we would be supportive of each other, and certainly if we have a difficult case we would talk about it very freely with my two colleagues, and I think that acts as a sort of a debriefing type of intervention in itself.”[GP1]

“… it has a big impact on how you deal with other patients in your day to day practice. Your concentration is not the same and you relive things and increased risk of mistakes and lack of care and lack of concentration so

For peer review only

you're not really giving your patients 100%. But I didn't seek help then, but my partners were brilliant, they were very supportive and they were very good and we talked about it a lot.[GP11]

GPs described how general practice can be "*reasonably isolated*." One reported that they felt the impact of a patient suicide was more damaging for them than for other mental health professionals such as psychiatry. The GP is seen as "*all things to all men*" [GP6], a person who can cure anything. There is the expectation in psychiatry that no matter how hard you try, you will lose patients in this way "*whereas in general practice we don't find it easy to accept failure.*" [GP6]. GPs are closer to the local community, have personal contacts and may be involved with care of other family members.

"you're not just involved with that person but you're involved with the whole family, and that's real general practice…" [GP7]

Perhaps the strongest message from this theme is that no GP reported that they used professional support.

To conclude, GPs undergo complicated experiences when they lose patients to suicide. They spend time in intense personal and professional reflection. They may feel that a patient was lost who did not need to be or conclude that they could not have helped. GPs are left with a profound and lasting sense of guilt, responsibility and failure. They may blame themselves for the death, they may be blamed by others. Mistakes may have been made. This is often channelled into taking action to try and ensure it does not happen again. Sometimes it does and the need for support increases. GPs know that they need support, but they don't feel they can access it. The age-old question remains, "who heals the healer?".

Discussion

For peer review only - http://bmjopen.bmj.com/site/about/guidelines.xhtml

367 Previous studies have examined the impact of patient suicide on psychiatric staff but fewer
368 studies have examined specifically GPs reactions. And none to the author's knowledge in
369 Northern Ireland to the depth that this study did. We sought to explore personal and
370 professional impacts, and support systems available for GPs who experienced patient suicide.
371 We identified four themes: personal reaction, professional impact, barriers to accessing
372 support and psychological support. The themes are similar to those uncovered in studies of
373 suicide impact on other medical professionals. In terms of personal reaction, experiencing a
374 period of rumination or *"great self-scrutiny"* as described by Saini et al.(5) with similar
375 personal and professional impacts. Guilt, blame, shock, anger, sadness and grief following
376 the suicide of a patient are commonly reported.(2) GP distress was noted by Foggin et al.(13)
377 and Saini et al.(5) The current study additionally indicates the considerable psychological
378 and organisational barriers to GP support following patient suicide. We noted a significant
379 dissonance between the GP self-image of solid imperturbability, and the distress caused by
380 patient suicide. The idealised estimation of a 'good' GP embodied in a *'hidden contract'*
381 develops in training and is reinforced over years but in practice but appears to fail in the long-
382 term as GPs gain experience and insight into when they need to seek help and form
383 mechanisms to cope with difficult days. A level of objectivity or detachment from the patient
384 is a form of self-protection that may appear sensible and attractive in training but deteriorates
385 over time. One GP contrasted the loss of a patient to suicide in general practice to that
386 experienced in psychiatry where there is a commonly held expectation that some patients will
387 die by suicide. Regular contact with high-risk patients increases this expectation. In primary
388 care where suicide is relatively rare a patient's suicide is more likely to undermine the
389 professional identity of GPs who work as individuals rather than part of a clinical team.

For peer review only - http://bmjopen.bmj.com/site/about/guidelines.xhtml

390 The "*hidden contract*" discourages GP help-seeking for psychological support. It is seen as a weakness. This fear of being diminished by loss of regard by others has been described in other studies of self-care and intra-professional support among GPs and other high visibility caring professions in which coping, self-reliance and resilience may be over-valued to the detriment of the individual.(22,23)

395 GPs describe a high-pressure environment with an excessive workload all swiftly managed in consecutive ten-minute appointments. In this context it is difficult to provide compassionate care which requires time to listen in order to understand. GPs are conflicted by systemic requirements for example they might wish to request hospital admission for people whose mental health has deteriorated knowing this is in opposition to the policy of community care. 400 Other role conflicts are the amount of time spent with individuals should be dictated by patients' needs but can't be due to time demands and pressures. GPs may want to acknowledge the distress of bereaved patients but seek to convey a detached professionalism.

Cheshire et al.(24) used a qualitative design to understand how GPs coped with workplace challenges and stresses. Among these were the inability to connect authentically due to time 405 demands and requirements such as the need to record patient indicators within the ten minutes appointment. GPs felt primary care was functioning in more detached ways which are more comparable with secondary care functioning. Some GPs felt that working longer hours was the most effective way they could deal with the sense of being overwhelmed, to the detriment of a work-life balance. Strategies which the GPs used to mitigate work stress 410 included meditation, mindfulness, stress management techniques, taking regular exercise, eating well and meeting and debriefing with their colleagues however the study also reported that a surprisingly high number of participants had adopted changes such as reducing their

For peer review only - http://bmjopen.bmj.com/site/about/guidelines.xhtml

working hours or changing their role to locum to allow them to downsize and regain work-life balance.(24)

Emotional hardening and cynicism among GP appeared ubiquitous. An example of why this may be was provided by one GP who described going to identify a body after a suicide and on their return to a busy day and a list of patients, "*no-one even asked*" how they were, suggesting a resigned fatalism for at least one GP in this sample. Perhaps exacerbating the self-neglect reported was that GPs were concerned about the professional repercussions that might accompany accessing support. GPs raised the issue of potentially being declared unfit to practise if formal help was sought, a finding supported by previous research. Spiers et al.(25) found that GPs reported issues around privacy, confidentiality and the stigma surrounding seeking help. This worry around professional repercussions begins with medical training and is reported by medical students.(26)

The lack of uptake of services for mental health issues in the wake of the loss resonates with low GP uptake of care when physically ill in a study reported by Thompson and colleagues.(23) GPs want help but don't feel they can access it. The impact of this inability to seek help is seen in studies conducted on the mental health of doctors. Doctor's experience high rates of anxiety,(27) depression,(28) substance misuse,(29) emotional exhaustion and burnout(30) and are at high risk of suicide themselves.(26) Male doctors are 1-4 times more likely than other men to die by suicide and female doctors 2-3 times more likely than other women.(31) This study showed that the impact of loses of this nature may even lead a GP to consider leaving the profession which has implications on a personal, practice level and regional service provision level.

For peer review only - http://bmjopen.bmj.com/site/about/guidelines.xhtml

GP uncertainty about the availability of support may indicate their own ambivalence about help-seeking or the inappropriateness of current available support. While GPs are seen as part of the National Health Service in the UK, they are individual practitioners who work as self-employed practitioners within GP surgeries. One review of the literature recommended that doctors require specialist services which focus on the unique and complex situation of a doctor becoming a patient.(32) George et al.(33) outline a pilot aimed at preventing or minimising mental health issues in doctors. This strategy would operate at a primary, secondary and tertiary level. Primary level prevention would be aimed mainly at medical students and may work to counteract the macho culture in which a doctor is expected to show no weakness. This level would include awareness-raising, enhancing coping skills and stress management skills. Secondary level prevention would allow early diagnosis and intervention and would include measures for screening, reducing stigma and creating a supportive culture for doctors. The tertiary level would include offering treatments and interventions to minimise the chronic negative effects of mental ill health on doctors.

Strengths of this study lie in the qualitative methodology employed in an areas where depth of experience is complicated, nuanced and important to understand. The study allowed GPs to talk in depth about a subject matter which is both stigmatised and sombre. This allowed responses to be accessed in an area not widely researched in a population not comfortable discussing this topic. Findings showed that GPs are not comfortable but nevertheless need to talk about this loss, and this study provided a forum for them to do that, one which would not normally be presented. Limitations include that findings cannot necessarily be extrapolated to other medical settings outside of primary care and are not representative worldwide as care systems vary and different systems will offer different responses. However there is bound to be something fundamentally similar in the reaction to this loss from someone who has

For peer review only

dedicated their life to helping others. To know rather than surmise that further GPs, psychiatrists, psychologists and other suicide professionals should be incorporated in future research to offer additional insight and understanding. It is important to note also that the emotional aspect of guilt and self-scrutiny following the death of a patient by suicide, may have contributed to potential under-reporting on the effects of patient suicide. Only by making the changes needed which will reduce the stigma around this issue can we know the scale as well as the nature of the impacts.

Conclusions

The findings of this study suggest a need to provide GPs with the appropriate psychological support after losing a patient to suicide. Despite a wish to remain true to the GP self-image of solid imperturbability created by adherence to the 'hidden contract', GPs report that the loss of a patient to suicide results in a period of self-scrutiny. From this may come short or longer term effects on their mental health. The "*hidden contract*" translates into doctors being unable to comfortably make a transition to patient which means that doctors are unable to properly receive the care they seek to provide for others.

Creating a culture shift which allows doctors to complete this transition will help prevent burnout and early retirement from a system which is not currently supporting them. One way to help create that change is to include an opposing view as part of training for all health and social care professionals for example by including in the curriculum modules on how to understand and deal with the effects of self-harm and suicide. The price for seeking that help should not include that their fitness to practice will be questioned consequently. GPs work in a high risk, high stress environment in which they are likely to experience loss which will require intervention and support. We recommend the provision of a strategy which will

For peer review only

facilitate the culture change needed and implement the services needed to support our doctors in general practice, including guidelines for GPs on when and how to ask for help. If a change is not facilitated, we will continue to see GPs experiencing distress and mental health issues which when unaddressed are contributing to mental illness, burnout and early retirement.

Contributors GMcA, KN and GL were responsible for data analysis and interpretations. GMcA wrote the initial draft of the article. GMcA revised the article in response to the comments of the co-authors after they commented critically on the draft. All authors gave their final approval of the version to be published.

Competing interests None declared.

Funding This study was funded by the Northern Ireland Public Health Agency (Grant COM/4032/08). The funder played no part in the conduct or reporting of the study, nor had any involvement in the interpretation of the data.

Data sharing statement No additional data available.

Figure 1: Thematic map showing relationship between themes

For peer review only

References

1. Leavey, G., Rosato, M., Galway, K., Hughes, L., Mallon, S., & Rondon, J. (2016). Patterns and predictors of help-seeking contacts with health services and general practitioner detection of suicidality prior to suicide: a cohort analysis of suicides occurring over a two-year period. *BMC psychiatry*, *16*, 1-8.

2. Sandford, D. M., Kirtley, O. J., Thwaites, R., & O'Connor, R. C. (2020). The impact on mental health practitioners of the death of a patient by suicide: a systematic review. In *Clinical Psychology & Psychotherapy* (Vol. 2). https://doi.org/10.1002/cpp.2515

3. Leavey, G., Mallon, S., Rondon-Sulbaran, J., Galway, K., Rosato, M., & Hughes, L. (2017). The failure of suicide prevention in primary care: family and GP perspectives–a qualitative study. *BMC psychiatry*, *17*(1), 1-10.

4. Pavlič, D. R., Treven, M., Maksuti, A., Švab, I., & Grad, O. (2018). General practitioners' needs for support after the suicide of patient: A qualitative study. *European Journal of General Practice*, *24*(1), 177–182. https://doi.org/10.1080/13814788.2018.1485648

5. Saini, P., Chantler, K., While, D., & Kapur, N. (2016). Do GPs want or need formal support following a patient suicide?: A mixed methods study. *Family Practice*, *33*(4), 414–420. https://doi.org/10.1093/fampra/cmw040

6. Alexander, D. A., Klein, S., Gray, N. M., Dewar, I. G., & Eagles, J. M. (2000). Suicide by patients: Questionnaire study of its effect on consultant psychiatrists. *British Medical Journal*, *320*(7249), 1571–1574. https://doi.org/10.1136/bmj.320.7249.1571

7. Murphy, P. T., Clogher, L., Van Laar, A., O'Regan, R., McManus, S., McIntyre, A., O'Connell, A., Geraghty, M., Henry, G., & Hallahan, B. (2019). The impact of service

For peer review only - http://bmjopen.bmj.com/site/about/guidelines.xhtml

user's suicide on mental health professionals. *Irish Journal of Psychological Medicine*, *April 2020*. https://doi.org/10.1017/ipm.2019.4

8. Finlayson, M., & Graetz Simmonds, J. (2018). Impact of Client Suicide on Psychologists in Australia. *Australian Psychologist*, *53*(1), 23–32. https://doi.org/10.1111/ap.12240

9. Gibbons, R., Brand, F., Carbonnier, A., Croft, A., Lascelles, K., Wolfart, G., & Hawton, K. (2019). Effects of patient suicide on psychiatrists: survey of experiences and support required. *BJPsych Bulletin*, *43*(5), 236–241. https://doi.org/10.1192/bjb.2019.26

10. Gaffney, P., Russell, V., Collins, K., Bergin, A., Halligan, P., Carey, C., & Coyle, S. (2009). Impact of patient suicide on front-line staff in Ireland. *Death Studies*, *33*, 639-656.

11. Foley, S. R., & Kelly, B. D. (2007). When a patient dies by suicide: Incidence, implications and coping strategies. *Advances in Psychiatric Treatment*, *13*(2), 134–138. https://doi.org/10.1192/apt.bp.106.002501

12. Zambrano, S. C., & Barton, C. A. (2011). On the journey with the dying: how general practitioners experience the death of their patients. *Death Studies*, *35*(9), 824–851.

13. Foggin, E., McDonnell, S., Cordingley, L., Kapur, N., Shaw, J., & Chew-Graham, C. A. (2016). GPs' experiences of dealing with parents bereaved by suicide: A qualitative study. *British Journal of General Practice*, *66*(651), e737–e746. https://doi.org/10.3399/bjgp16X686605

14. Halligan, P., & Corcoran, P. (2001). The impact of patient suicide on rural general practitioners. *British Journal of General Practice*, *51*(465), 295–296.

For peer review only - http://bmjopen.bmj.com/site/about/guidelines.xhtml

15. Leavey, G., Galway, K., Hughes, L., Mallon, S., Sulbarán, J. R., & Rosato, M. (2016). Understanding Suicide and Help-Seeking in Urban and Rural Areas in Northern Ireland. *Bamford Centre for Mental Health and Wellbeing, Ulster University: Belfast, UK*.

16. Braun, V., & Clarke, V. (2006). Using thematic analysis in psychology. *Qualitative research in psychology*, *3*, 77-101.

17. Smith, J. A., Flowers, P., & Larkin, M. (2013). *Interpretative Phenomenological Analysis: Theory, Method and Research. London*: SAGE Publications. https://doi.org/10.1080/14780880903340091

18. Larkin, M., Watts, S., & Clifton, E. (2006). Giving voice and making sense in interpretative phenomenological analysis. Qualitative Research in Psychology, 3(2), 102-120. Retrieved from ttps://www.tandfonline.com/doi/abs/10.1191/1478088706qp062oa

19. Ormston, R., Spencer, L., Barnard, M., & Snape, D. (2014). The foundations of qualitative research. *Qualitative research practice: A guide for social science students and researchers*, *2*, 52-55. Retrieved from http://www.jbposgrado.org/icuali/Qualitative%20Research%20practice.pdf

20. Savin-Baden, M., & Major, C. H. (2013). Qualitative research: The essential guide to theory and practice. https://www.routledge.com/Qualitative-Research-The-essential-guide-to-theory-and-practice-1st-Edition/Savin-Baden-Howell-Major/p/book/9780415674782.

For peer review only - http://bmjopen.bmj.com/site/about/guidelines.xhtml

21. O'Brien, B. C., Harris, I. B., Beckman, T. J., Reed, D. A., & Cook, D. A. (2014). Standards for reporting qualitative research: a synthesis of recommendations. *Academic medicine*, *89*(9), 1245-1251.

22. Leavey, G. (2008). UK clergy and people in mental distress: Community and patterns of pastoral care. *Transcultural Psychiatry*, *45*, 79-104.

23. Thompson, W. T., Cupples, M. E., Sibbett, C. H., Skan, D. I., & Bradley, T. (2001). Challenge of culture, conscience, and contract to general practitioners' care of their own health: qualitative study. *BMJ: British Medical Journal*, *323*(7315), 728.

24. Cheshire, A., Ridge, D., Hughes, J., Peters, D., Panagioti, M., Simon, C., & Lewith, G. (2017). Influences on GP coping and resilience: a qualitative study in primary care. *British Journal of General Practice*, *67*, e428-e436.

25. Spiers, J., Buszewicz, M., Chew-Graham, C. A., Gerada, C., Kessler, D., Leggett, N., ... & Riley, R. (2017). Barriers, facilitators, and survival strategies for GPs seeking treatment for distress: a qualitative study. *British Journal of General Practice*, *67*, e700-e708.

26. Devi, S. (2011). Doctors in distress. *The Lancet*, *377*(9764), 454-455.

27. Kroenke, K., Spitzer, R. L., Williams, J. B., Monahan, P. O., & Löwe, B. (2007). Anxiety disorders in primary care: prevalence, impairment, comorbidity, and detection. *Annals of internal medicine*, *146*, 317-325.

28. Firth-Cozens, J. (2001). Interventions to improve physicians' well-being and patient care. *Social science & medicine*, *52*, 215-222.

For peer review only - http://bmjopen.bmj.com/site/about/guidelines.xhtml

29. Bennett, J., & O'Donovan, D. (2001). Substance misuse by doctors, nurses and other healthcare workers. *Current opinion in Psychiatry*, *14*, 195-199.

30. Rø, K. E. I., Gude, T., Tyssen, R., & Aasland, O. G. (2008). Counselling for burnout in Norwegian doctors: one year cohort study. *BMJ*, *337*.

31. Schernhammer, E. S., & Colditz, G. A. (2004). Suicide rates among physicians: a quantitative and gender assessment (meta-analysis). *American Journal of Psychiatry*, *161*, 2295-2302.

32. Brooks, S. K., Gerada, C., & Chalder, T. (2011). Review of literature on the mental health of doctors: are specialist services needed?. *Journal of Mental Health*, *20*, 146-156.

33. George, S., Deshpande, S., & Kallivayalil, R. A. (2020). Are specialist treatment services needed for doctors with mental health problems?.*Indian Journal of Psychological medicine*, *42*, 105-107.

For peer review only

For peer review only - http://bmjopen.bmj.com/site/about/guidelines.xhtml

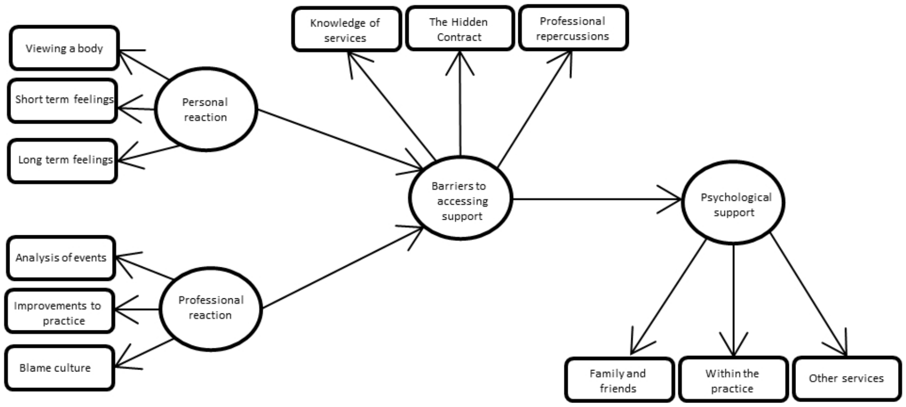

90x90mm (300 x 300 DPI)

Standards for Reporting Qualitative Research (SRQR)*

http://www.equator-network.org/reporting-guidelines/srqr/

Title and abstract

	Page/line no(s).
**Title** - Concise description of the nature and topic of the study Identifying the study as qualitative or indicating the approach (e.g., ethnography, grounded theory) or data collection methods (e.g., interview, focus group) is recommended	Title Page Lines 1&2
**Abstract** - Summary of key elements of the study using the abstract format of the intended publication; typically includes background, purpose, methods, results, and conclusions	Page 1 Lines 1-16

Introduction

**Problem formulation** - Description and significance of the problem/phenomenon studied; review of relevant theory and empirical work; problem statement	Pages 3-4 lines 32-68
**Purpose or research question** - Purpose of the study and specific objectives or questions	Page 4 lines 60-68

Methods

**Qualitative approach and research paradigm** - Qualitative approach (e.g., ethnography, grounded theory, case study, phenomenology, narrative research) and guiding theory if appropriate; identifying the research paradigm (e.g., postpositivist, constructivist/ interpretivist) is also recommended; rationale**	Page 4/5 lines 71-81
**Researcher characteristics and reflexivity** - Researchers' characteristics that may influence the research, including personal attributes, qualifications/experience, relationship with participants, assumptions, and/or presuppositions; potential or actual interaction between researchers' characteristics and the research questions, approach, methods, results, and/or transferability	Page 6/7 lines 108-119
**Context** - Setting/site and salient contextual factors; rationale**	Page 5 lines 88-97
**Sampling strategy** - How and why research participants, documents, or events were selected; criteria for deciding when no further sampling was necessary (e.g., sampling saturation); rationale**	Page 5 lines 88-97
**Ethical issues pertaining to human subjects** - Documentation of approval by an appropriate ethics review board and participant consent, or explanation for lack thereof; other confidentiality and data security issues	Page 5 lines 72-81
**Data collection methods** - Types of data collected; details of data collection procedures including (as appropriate) start and stop dates of data collection and analysis, iterative process, triangulation of sources/methods, and modification of procedures in response to evolving study findings; rationale**	Page 5 lines 88-97<br>Page 6 lines 99-106

For peer review only - http://bmjopen.bmj.com/site/about/guidelines.xhtml

**Data collection instruments and technologies** - Description of instruments (e.g., interview guides, questionnaires) and devices (e.g., audio recorders) used for data collection; if/how the instrument(s) changed over the course of the study	Pages 5/6 lines 88-97
**Units of study** - Number and relevant characteristics of participants, documents, or events included in the study; level of participation (could be reported in results)	Page 7 lines 132-136
**Data processing** - Methods for processing data prior to and during analysis, including transcription, data entry, data management and security, verification of data integrity, data coding, and anonymization/de-identification of excerpts	Page 6 Lines 99-75
**Data analysis** - Process by which inferences, themes, etc., were identified and developed, including the researchers involved in data analysis; usually references a specific paradigm or approach; rationale**	Page 5 lines 93-101
**Techniques to enhance trustworthiness** - Techniques to enhance trustworthiness and credibility of data analysis (e.g., member checking, audit trail, triangulation); rationale**	Page 7 Lines 120-129

Results/findings

**Synthesis and interpretation** - Main findings (e.g., interpretations, inferences, and themes); might include development of a theory or model, or integration with prior research or theory	Pages 7-17 Lines 132- 363
**Links to empirical data** - Evidence (e.g., quotes, field notes, text excerpts, photographs) to substantiate analytic findings	Pages 7-17 Lines 132- 363

Discussion

**Integration with prior work, implications, transferability, and contribution(s) to the field -** Short summary of main findings; explanation of how findings and conclusions connect to, support, elaborate on, or challenge conclusions of earlier scholarship; discussion of scope of application/generalizability; identification of unique contribution(s) to scholarship in a discipline or field	Pages 18-22 Lines 364-445
**Limitations** - Trustworthiness and limitations of findings	Page 1/2 Lines 17-29<br>Page 22 Lines 446-462

Other

**Conflicts of interest** - Potential sources of influence or perceived influence on study conduct and conclusions; how these were managed	Page 24 line 490
**Funding** - Sources of funding and other support; role of funders in data collection, interpretation, and reporting	Page 24 Lines 491-493

*The authors created the SRQR by searching the literature to identify guidelines, reporting standards, and critical appraisal criteria for qualitative research; reviewing the reference lists of retrieved sources; and contacting experts to gain feedback. The SRQR aims to improve the transparency of all aspects of qualitative research by providing clear standards for reporting qualitative research.

For peer review only

For peer review only - http://bmjopen.bmj.com/site/about/guidelines.xhtml

**The rationale should briefly discuss the justification for choosing that theory, approach, method, or technique rather than other options available, the assumptions and limitations implicit in those choices, and how those choices influence study conclusions and transferability. As appropriate, the rationale for several items might be discussed together.

Reference:

O'Brien BC, Harris IB, Beckman TJ, Reed DA, Cook DA. **Standards for reporting qualitative research: a synthesis of recommendations.** *Academic Medicine*, Vol. 89, No. 9 / Sept 2014 DOI: 10.1097/ACM.0000000000000388

For peer review only

BMJ Open

A qualitative study investigating the professional and personal effects of patient suicide on General Practitioners in Northern Ireland.

Journal:	*BMJ Open*
Manuscript ID	bmjopen-2023-077940.R2
Article Type:	Original research
Date Submitted by the Author:	22-Jan-2024
Complete List of Authors:	McAnee, Grainne; Ulster University, School of Psychology<br>Norwood, Kelly; Ulster University Faculty of Life and Health Sciences, School of Psychology<br>Leavey, Gerard; University of Ulster, Psychology
**Primary Subject Heading**:	General practice / Family practice
Secondary Subject Heading:	Qualitative research, Mental health
Keywords:	MENTAL HEALTH, MEDICAL EDUCATION & TRAINING, Burnout, Professional, GENERAL MEDICINE (see Internal Medicine)

SCHOLARONE™
Manuscripts

I, the Submitting Author has the right to grant and does grant on behalf of all authors of the Work (as defined in the below author licence), an exclusive licence and/or a non-exclusive licence for contributions from authors who are: i) UK Crown employees; ii) where BMJ has agreed a CC-BY licence shall apply, and/or iii) in accordance with the terms applicable for US Federal Government officers or employees acting as part of their official duties; on a worldwide, perpetual, irrevocable, royalty-free basis to BMJ Publishing Group Ltd ("BMJ") its licensees and where the relevant Journal is co-owned by BMJ to the co-owners of the Journal, to publish the Work in this journal and any other BMJ products and to exploit all rights, as set out in our licence.

The Submitting Author accepts and understands that any supply made under these terms is made by BMJ to the Submitting Author unless you are acting as an employee on behalf of your employer or a postgraduate student of an affiliated institution which is paying any applicable article publishing charge ("APC") for Open Access articles. Where the Submitting Author wishes to make the Work available on an Open Access basis (and intends to pay the relevant APC), the terms of reuse of such Open Access shall be governed by a Creative Commons licence – details of these licences and which Creative Commons licence will apply to this Work are set out in our licence referred to above.

Other than as permitted in any relevant BMJ Author's Self Archiving Policies, I confirm this Work has not been accepted for publication elsewhere, is not being considered for publication elsewhere and does not duplicate material already published. I confirm all authors consent to publication of this Work and authorise the granting of this licence.

For peer review only

Abstract

Objective: There is a dearth in suicide literature addressing the impact on general practitioners of losing a patient. We aimed to examine the personal and professional impact as well as the availability of support and why GPs did or did not use it.

Design: A qualitative study using one to one interviews with participants recruited using snowball sampling.

Setting: The study was conducted in a primary care setting.

Participants: Interviews were held with 19 GPs within primary care in Northern Ireland.

Results: GPs are impacted both personally and professionally when they lose a patient to suicide, but may not access formal help due to commonly-held idealised notions of a “good” GP who is regarded as having solid imperturbability. Fear of professional repercussions also plays a major role in deterring help-seeking.

Conclusions: There is a need for a systemic culture shift within general practice which allows doctors to seek support when their physical or mental health require it. This may help prevent stress, burnout and early retirement.

Strengths and limitations of this study

- The qualitative methodology employed in this study allows the depth of experience to be accessed in an area not widely researched in a population not comfortable discussing this topic.

For peer review only

- The study explored experiences of patient suicide within the context of primary care in NI, therefore these findings cannot be extrapolated to other medical settings and are not representative worldwide.
- For a more inclusive picture, further GPs, psychiatrists, psychologists, nurses, community pharmacists and other professionals should be incorporated in the research to offer additional insight and understanding.
- The emotional aspect of guilt and self-scrutiny following the death of a patient by suicide, may have contributed to potential under-reporting on the effects of patient suicide.

For peer review only

For peer review only - http://bmjopen.bmj.com/site/about/guidelines.xhtml

A qualitative study investigating the professional and personal effects of patient suicide on General Practitioners in Northern Ireland.

Introduction

It estimated up to 90% of people who die by suicide consult their GP shortly before death, with 20% - 76% seeing their GP in the month prior.(1) Impacts on GPs are both personal and professional.(2) Understanding these impacts may facilitate the implementation of support needed to allow GPs to understand and recover from patient suicide- induced trauma. There is a dearth in suicide literature addressing the reactions of GPs to this loss.(3-5)

A 2020 systematic review assessed the impact on mental health practitioners when they experienced this loss.(2) Guilt, blame, shock, anger, sadness and grief were common(2), in addition to lowered mood, poor sleep and increased irritability.(6) Professional reactions included doubt about practice and decision-making,(7) more caution in the management of suicide risk(8) and consideration of retirement or career change.(9) Similar research investigated the effects on frontline staff reporting that staff questioned themselves wondering if they could have helped more.(10)

Studies have examined the effects on psychiatrists and psychologists(11) with some focussing on GPs. In Pavlič et al.'s qualitative study of Slovenian GP experiences (n=22) GPs needed emotional support after a patient suicide but reported barriers including lack of provision and personal hesitation due to perceptions of weakness and professional failure - leading GPs to only seek support from close colleagues.(4) Zambrano and Barton(12) reported that GP coping mechanisms included speaking with colleagues and acceptance of the death as unavoidable. A UK based study using 198 semi-structured interviews with GPs noted a lack of institutional understanding and poor professional support.(5)

For peer review only

For peer review only - http://bmjopen.bmj.com/site/about/guidelines.xhtml

Another UK based study focused on GPs experiences of dealing with parents bereaved by suicide while considering the impact on GPs.(13) They described personal grief at losing a patient for whom they cared. The stigma of GPs accessing formal support and reliance on colleague support were consistent themes. A survey of 152 rural GPs in the Republic of Ireland(14) who lost patients who died by suicide revealed GP reactions included feelings of guilt, sleep disruption, increased readiness to make psychiatric referrals in patient care and reduced confidence in decision making.

In summary, GPs experiences of suicide affect them on a personal level and inform how they care for future patients at risk of suicide. This study adds to the literature addressing how GPs react to the suicide of patients by exploring the impact at a personal and professional level using interviews with nineteen GPs in Northern Ireland. To the best of the author's knowledge this is the first qualitative study of this scale in Northern Ireland. It is one which explored access to support in this population in depth. Finally, it allows the needs of GPs in Northern Ireland to be better responded to. It asked two main questions: What was the personal and professional impact of patient suicide on GPs? What was the availability of support and the reasons for access or avoidance?

Method

Design

The study 'Understanding Suicide and Help-Seeking in Urban and Rural Areas in Northern Ireland'(15) received ethical approval from the Office of Research Ethics Committees Northern Ireland (REC reference: 10/NIR03/65) and the main report provides full details of the methodology. This qualitative study employed in-depth interviews (n=19) which were

For peer review only

recorded and transcribed to gain an understanding of the impact of patient suicide on practising GPs, support that was available and support needed. Informed consent was given after comprehensive information on the topic was shared with potential interviewees. Experienced researchers were employed with interpersonal skills suited to the topic, should interviewees become distressed during interviews they were reminded they were allowed to stop the interview at any time should they find it too upsetting.

Patient and public involvement

The project was supported by a Patient and Public Involvement (PPI) committee from its conception through to conclusion. The committee advised on all stages of the project. This included formulation of the research questions, design, recruitment and how the findings would be disseminated.

Participants

The full study within which this is placed(15) interviewed both family members of people in Northern Ireland who died by suicide and GPs. GPs who were working in NI and experienced the suicide of at least one patient in their care were invited to participate in the research by letter, with a follow-up phone call, according to the geographical location of their practice in order to represent any disparities between urban and rural areas. After a number of unsuccessful attempts to recruit GPs through traditional postal invitations, the 19 GPs who participated in the study were recruited through a snowball sampling approach with support from the Royal College of General Practitioners (NI). The study explored: (1) contact, recognition and management of suicidal patient; (2) primary care liaison with psychiatric and other services; and (3) recommendations for improved care and suicide prevention

Procedure and Data analysis

For peer review only

99 Qualitative data from interviews were analysed using inductive, thematic analysis via
100 NVivo12. Data were analysed via a six-phase process: data familiarisation, generating initial
101 codes, searching for themes, reviewing the themes, defining and naming the themes and
102 report writing.(16) Three researchers (KN, GMA, AD) read transcripts twice for
103 familiarisation, then independently generated initial codes. Researchers independently coded
104 transcripts in batches of 5 and then met to review and collapse codes. All transcripts were re-
105 analysed using the final set of agreed codes from the iterative process. From these themes and
106 sub-themes were generated and reviewed until consensus was reached.

107 Reflexivity

108 Qualitative researchers are required to make sense of the material they engage with(17) and
109 as such it is imperative that they acknowledge how their own experiences and beliefs can
110 impact their perspective. With such an emotive and delicate topic it was important to be
111 mindful and take appropriate steps against this. Participants provided data on experiences
112 which are powerful and important as well as deeply personal and the respect it was treated
113 with included that it was approached with sensitivity and objectivity. It was important that
114 researchers remain open to the possibility of findings being contradictory to personal feelings
115 and beliefs.(18) Qualitative research can involve the practice of analysts disregarding prior
116 knowledge and experience(19) however this may not always be possible or indeed necessary.
117 (17) The researchers adopted a practice of self-reflection and consensus of three researchers
118 to protect robustness of findings. The use of a literature review before analysis and placing
119 findings within the context of that literature also protected the integrity of analysis.

120 Trustworthiness and credibility

For peer review only - http://bmjopen.bmj.com/site/about/guidelines.xhtml

Due to lack of standardisation of the methodologies employed in qualitative research it is not possible to conduct formal reliability testing however Savin-Baden and Major(20) provide criteria to improve trustworthiness which include methodological coherence (congruence between research objectives, methodologies, data and analysis), audit trail (description of the research process from beginning to end which can uncover other possible influences on findings), and negative case analysis (actively identifying and extracting data which was not compatible with the main or expected findings in order to encourage critical thinking and subsequent modification). The use of the Standards for Reporting Qualitative Research (SRQR) was adopted to further the aim of trustworthiness.(21)

Results

Nineteen GPs took part (n=11 women and n=8 men) from a range of primary care settings, (socio-economic and urban, semi-urban and rural populations). Participants were long-serving practitioners (15-30 years' experience), involved in the care of more than 2 patients who died by suicide, several GPs indicated more than 10 cases, and three had personal experiences of suicide.

Analysis of the data generated four themes; personal reaction, professional impact, barriers to accessing support and psychological support. Themes and sub-themes are shown in Table 1, Figure 1 shows the relationships between them.

For peer review only - http://bmjopen.bmj.com/site/about/guidelines.xhtml

Table 1: Summary of Themes and Sub-Themes Generated from GP Interviews

Themes	Sub-themes
1. Personal reaction	Viewing a body.<br>Short term feelings.<br>Long term feelings.
2. Professional impact	Analysis of events<br>Improvements to practice.<br>Blame culture.
3. Barriers to accessing support	Knowledge of services.<br>The hidden contract.<br>Professional repercussions.
4. Psychological support	Family and friends .<br>Within the practice.<br>Other services.

[Figure 1 here]

Most GPs had been in practice for more than 20 years with knowledge of multiple suicides, in practice and in their personal lives. GPs had experienced the loss of siblings, other family members, friends and colleagues. They supported family members of patients, close

For peer review only

For peer review only - http://bmjopen.bmj.com/site/about/guidelines.xhtml

colleagues and other members of their practice. The impact of a suicide was variously described as '*devastating'* or '*very painful*'. No GP was unaffected by the suicide of a patient and effects were negative. Several GPs became distressed during interviews.

Five patient suicide typologies were interwoven into the experiences recounted: (1) Avoidable deaths: Regarded by GPs as people who might still be alive had services acted more swiftly or were better coordinated; (2) Missed signs: Suicides signalled by the patient but the GP recognised only in hindsight. (3) Valued patients: Suicide of patients who had coped with chronic illness with cheerful stoicism; (4) Apparent improvement: Suicides that occur despite apparent good mood and social functioning; (5) Family complaint: GP feels scapegoated by the family.

Personal Reaction

Most GPs reported that the suicide of a patient "*has a massive impact on the clinician as a person*" [GP11]. The work of the average GP means that they will encounter what one described as '*endless human misery*', with a GP describing the impact:

> *"Your whole life is absorbing. … you absorb everything … everybody's pain and sorrow …"[GP9]*

Feelings expressed by GPs when they lost a patient included shock, loss, anger, upset, distress, desperation and sadness. The strongest feelings were responsibility, guilt and failure, "*there is an awful lot of guilt and everybody feels guilty*"[GP12]. Several GPs said they had experienced periods of depression and had witnessed colleagues being "*destroyed*" in the aftermath.

For peer review only - http://bmjopen.bmj.com/site/about/guidelines.xhtml

GPs expressed that they found the process of confirming the death to be "*very, very troubling*" [GP2] and in a manner resonating with post-traumatic stress disorder, described how it became "*a very visual memory*" [GP2] which remained with them. After this initial reaction came short term feelings. One GP talked about the suicide of an elderly patient who had started exhibiting erratic behaviour. The patient was engaged with services at the time with professionals disagreeing over a diagnosis. The GP felt the patient had fallen into "*the gaps of the system*" [GP11]. The short-term impact on the GP was devastating.

> *"I came in and did my work and I went home and I cried, probably for about two weeks, all night." [GP11]*

Short terms feelings were led by a period of intense reflection after the loss during which they ruminated on the events preceding the suicide because "*there is always a huge guilt issue around suicide*." [GP7]. One GP sums up this dynamic and the need for reassurance.

> *"You want to know it's not your fault. You feel guilty because there's always something you could do… or maybe there isn't but you always feel that." [GP4]*

Different conclusions were reached seeking to quiet that voice that suggested that "*there's always something you can do.*" [GP1]. These depended on assessment of prior events and relationships between the patient, GP, and other services. Where there had been a history of self-harm, alcohol or substance abuse, poor engagement with services or impulsive behaviour, GPs indicated that these deaths were upsetting but not shocking and they could not have changed the outcome; "*when you reflect on what could you have done differently that might have had an impact, most of them you couldn't.*"[GP5] This recognition did not always permit feelings of failure to be resolved.

For peer review only

Other GPs concluded the outcome could have been changed. One GP working in an area of high deprivation reflected on the death of a young patient with a history of psychiatric problems who had been seen by several services in the days leading to their suicide. The patient stated they were suicidal and had shown signs of being suicidal during a home visit. Although services were alerted, the patient died by suicide. The GP felt that the system had failed.

"I was very upset about it because I felt that if he had been immediately sectioned he would have been alive..."[GP1]

Others had the anxiety of never knowing if alternative action could have altered the outcome. One GP talks about patients who "*leave no footprints in the snow*" [GP19]. This GP is describing patients who show no signs of considering suicide and leads the GP to wonder both,

"is it a switch that can flick in your head? And the natural follow-up from that is could that flick in anybody's head…"[GP19]

The description used is suggestive of something very haunting with a sense of silence wrapped around it. Another GP believed that,

"…most people who end up committing suicide, there probably is something documented … about their mood."[GP14]

One GP described the experience of losing a young person who had previously presented with suicidal ideation. Although in treatment they showed no signs of being actively suicidal; they regularly attended the practice but consultations were about minor physical issues. The loss of the patient "*was not a predicted suicide"* [GP12] and it "*really shocked the whole*

For peer review only

practice." [GP12]. Hindsight revealed signs that were not recognised and they were left feeling that "*in retrospect maybe things were more difficult."* [GP12] than the patient suggested. These questions remained unresolved, leaving feelings of guilt.

One difficultly expressed by GPs was the non-return of a patient whose mental state was concerning. GPs may assume that the problem is resolved, for patients the opposite may be true.

> *"I suppose as a GP you always assume that patients don't come back to see you if they're better, and that often is not the case, especially with mental health." [GP3]*

Part of the reason that GPs do not follow up is due to the nature and management of their workload. GPs describe an overloaded day with little room to spend time with patients and none for activities like following up patients they may have had a concern about; a 'niggle'. GPs do not know if they could have prevented a suicide but they also don't know the optimal criteria for intervention, how or when to intrude - "*you're never going to find out*" [GP4]. It can be very hard to know when the steps taken saved a life, but they will always know when they did not.

Long term feelings sometimes meant that even years after, the suicide was experienced as a painful memory. One GP described the "*intensely harrowing*" experiences of a close colleague who had experienced several "*traumatic suicides*", as a "*thinly covered scar*" [GP6] implying a wound that has never really healed. Another long-time impact is when other suicides occur "*it brings back the memories then of the others"* [GP7].

Professional Impact

For peer review only

There was a professional impact of a patient suicide. For most these effects provided the catalyst to change the practice management of suicidality, but several described how patients' suicide had provoked early retirement of colleagues. We noted considerable differences in how the aftermath was managed. Some practices had standard procedures such as conducting an event analysis to see "*whether we could have done anything differently or we could change our system*" [GP14] or meeting with other professionals involved for reviews. Even if there was no formal procedure, GPs performed some level of analysis. A GP who had experienced the death of their elderly patient described how they reviewed their notes with "*a fine-tooth comb*" [GP11]. These processes allowed GPs to explore and put in context the events leading up to the suicide.

There are complicated personal and social factors that influence GPs judgement and decision-making. In relation to risk management, one such factor was knowing the full picture. Some GPs became aware patients were telling families a different version of events than in consultations. One GP described how a family member was given "*a very different picture"* [GP12] compared to the GP around the patient's state of mind and what help had been both offered and rejected by the patient. Another GP revealed that they may have "*a lot of people pretending to be mentally ill to get disability living allowance*" [GP9].

A commonly reported impact was heightened vigilance.

> *"I would say I am much more vigilant and I'm now not the little kind of shy GP who is afraid to ask questions." [GP7]*

This extended to risk factors outside of the patient themselves such as a previous suicide in the family. It was not restricted to patients who presented with mental health issues, for example, it could be extended to patients in the same age range as the patient who had died.

For peer review only

> *"if I have a younger person in consultation now, I am looking at the mental health issues … prior history of any mental health issues" [GP12]*

Commonly expressed and contributing to feelings of guilt and responsibility was 'blame culture' - GPs felt they were being held accountable for patients' ill-health and their 'failure' to cure people often resulted in blame. GPs found this demoralising and professionally devaluing.

> *"People with no medical education, people with too much medical education, but everyone loves bashing GPs" [GP4]*

One GP who treated a young man shared the experience of being blamed when he died. The parents openly censured the GP, reproaching them for not doing more. The GP felt the parents had unrealistic expectations of primary care services and the extent of help available:

> *"… there was huge blame. The parents felt that we should have gone to their house and done more."[GP6]*

Barriers to accessing support

Given the impact described above it was worrying that GPs reported concerns and described barriers to accessing formal support. Three sub-themes were generated. Firstly, differing knowledge between GPs of available services. Some believed support by occupational health was available, others were sure it wasn't. One reason that GPs did not fully understand what help is available to them professionally is because they would be highly unlikely to consider using it. Even when there was knowledge about support services available, GPs stated it would not be accessed.

For peer review only

For peer review only - http://bmjopen.bmj.com/site/about/guidelines.xhtml

> *"The BMA have a good counselling service by telephone … it never occurred to me to use it."[GP6]*

A second sub-theme was that for GPs it is not acceptable to admit that sometimes those who help others, need to be helped themselves.

> *"GPs don't go sick. They work through ridiculous things because there's this macho "we're tough." "[GP6]*

An over-valued work ethic or sense of duty was referred to as "*the hidden contract*" or "*the hidden curriculum*" - described as an understanding that "*I will not tell you if I feel bad and I will not ask you if you feel bad*". It is a cultural concept that is absorbed during training. It appears to be strongly associated to an internalised perception of the GP as a healer, a source of strength, but not someone who may need support and care. While at one level this appears to be a deeply held cultural trope, on a more direct level, GP mental health issues may impinge on the doctor's fitness to practice.

In the final sub-theme we noted concerns related to professional repercussions if a GP accessed help related to their own mental health and/or ability to cope. They worry that their fitness to practice (and their career) will be jeopardised.

> *"…if you had a sore knee, that wouldn't be an issue; but if you admit to depression there is a big question mark about your fitness to practise."[GP10]*

The GPs overwhelmingly indicated they would not consider using support but they also said they knew it was needed;

For peer review only

For peer review only - http://bmjopen.bmj.com/site/about/guidelines.xhtml

"we need more support when suicides have happened… it needs to be built into the culture that we accept things when these things happen." [GP6]

Psychological Support

Given the intensity of GP's responses to losing a patient to suicide and the barriers reported above, the next question becomes centred on what support was available in assisting doctors to process the experiences and reduce negative impacts on future practice or potential premature retirement. If a GP does decide they would like support, instead of a robust and easily accessible GP support system it appears that the health system makes scant allowance for the impact of a traumatic event on the GP; most participants suggested that they are obliged to just get on with it.

"…but pull up your socks and get on with it. This is your job; get on with it. You have to deal with this."[GP11]

There was a strong message that it is hard for GPs to seek professional help for this loss and a sense of conflict around it.

"I'm not saying GPs don't need it, I'm saying that GPs don't access it, and the reason I think they don't access it is twofold. I suppose GPs feel that I'm big enough… I'm strong enough, I don't need counselling. And second of all, there's an embarrassment issue of having to seek help yourself as someone who gives help to others. I think there is a difficulty there." [GP1

For peer review only

For peer review only - http://bmjopen.bmj.com/site/about/guidelines.xhtml

GPs stated a lack of concern or compassion for them in the immediate aftermath, one describing an expectation that they would be able to continue working with no reaction to what they describe as a traumatic event,

> *"there's a huge difference in terms of hearing that someone has taken their own life and then being the GP who's actually going out to confirm that person has passed away, and I think it's one of the things, that it is fairly traumatic to see the person in that situation."[GP2]*

They found this expectation uncaring and resented it,

> *"The thing is we came back and just worked the rest of the shift… I just remember coming in the next day and no-one even saying, "that must have been tough.""[GP2]*

The most often used support was informal. Some GPs found support within their own homes, for example married GPs found support from their domestic partner, but for most GPs they found support within their own practice.

> *"I suppose in my practice we have three GPs. We are all much of the same age group and we would be supportive of each other, and certainly if we have a difficult case we would talk about it very freely with my two colleagues, and I think that acts as a sort of a debriefing type of intervention in itself."[GP1]*

> *"… it has a big impact on how you deal with other patients in your day to day practice. Your concentration is not the same and you relive things and increased risk of mistakes and lack of care and lack of concentration so*

For peer review only

you're not really giving your patients 100%. But I didn't seek help then, but my partners were brilliant, they were very supportive and they were very good and we talked about it a lot.[GP11]

GPs described how general practice can be "*reasonably isolated*." One reported that they felt the impact of a patient suicide was more damaging for them than for other mental health professionals such as psychiatry. The GP is seen as "*all things to all men*" [GP6], a person who can cure anything. There is the expectation in psychiatry that no matter how hard you try, you will lose patients in this way "*whereas in general practice we don't find it easy to accept failure.*" [GP6]. GPs are closer to the local community, have personal contacts and may be involved with care of other family members.

"you're not just involved with that person but you're involved with the whole family, and that's real general practice…" [GP7]

Perhaps the strongest message from this theme is that no GP reported that they used professional support.

To conclude, GPs undergo complicated experiences when they lose patients to suicide. They spend time in intense personal and professional reflection. They may feel that a patient was lost who did not need to be or conclude that they could not have helped. GPs are left with a profound and lasting sense of guilt, responsibility and failure. They may blame themselves for the death, they may be blamed by others. Mistakes may have been made. This is often channelled into taking action to try and ensure it does not happen again. Sometimes it does and the need for support increases. GPs know that they need support, but they don't feel they can access it. The age-old question remains, "who heals the healer?".

Discussion

For peer review only - http://bmjopen.bmj.com/site/about/guidelines.xhtml

Previous studies have examined the impact of patient suicide on psychiatric staff but fewer studies have examined specifically GPs reactions. And none to the author's knowledge in Northern Ireland to the depth that this study did. We sought to explore personal and professional impacts, and support systems available for GPs who experienced patient suicide. We generated four themes: personal reaction, professional impact, barriers to accessing support and psychological support. The themes are similar to those uncovered in studies of suicide impact on other medical professionals. In terms of personal reaction, experiencing a period of rumination or "*great self-scrutiny*" as described by Saini et al.(5) with similar personal and professional impacts. Guilt, blame, shock, anger, sadness and grief following the suicide of a patient are commonly reported.(2) GP distress was noted by Foggin et al.(13) and Saini et al.(5) The current study additionally indicates the considerable psychological and organisational barriers to GP support following patient suicide. We noted a significant dissonance between the GP self-image of solid imperturbability, and the distress caused by patient suicide. The idealised estimation of a 'good' GP embodied in a '*hidden contract*' develops in training and is reinforced over years but in practice but appears to fail in the long-term as GPs gain experience and insight into when they need to seek help and form mechanisms to cope with difficult days. A level of objectivity or detachment from the patient is a form of self-protection that may appear sensible and attractive in training but deteriorates over time. One GP contrasted the loss of a patient to suicide in general practice to that experienced in psychiatry where there is a commonly held expectation that some patients will die by suicide. Regular contact with high-risk patients increases this expectation. In primary care where suicide is relatively rare a patient's suicide is more likely to undermine the professional identity of GPs who work as individuals rather than part of a clinical team.

For peer review only - http://bmjopen.bmj.com/site/about/guidelines.xhtml

The "*hidden contract*" discourages GP help-seeking for psychological support. It is seen as a weakness. This fear of being diminished by loss of regard by others has been described in other studies of self-care and intra-professional support among GPs and other high visibility caring professions in which coping, self-reliance and resilience may be over-valued to the detriment of the individual.(22,23)

GPs describe a high-pressure environment with an excessive workload all swiftly managed in consecutive ten-minute appointments. In this context it is difficult to provide compassionate care which requires time to listen in order to understand. GPs are conflicted by systemic requirements for example they might wish to request hospital admission for people whose mental health has deteriorated knowing this is in opposition to the policy of community care. Other role conflicts are the amount of time spent with individuals should be dictated by patients' needs but can't be due to time demands and pressures. GPs may want to acknowledge the distress of bereaved patients but seek to convey a detached professionalism.

Cheshire et al.(24) used a qualitative design to understand how GPs coped with workplace challenges and stresses. Among these were the inability to connect authentically due to time demands and requirements such as the need to record patient indicators within the ten minutes appointment. GPs felt primary care was functioning in more detached ways which are more comparable with secondary care functioning. Some GPs felt that working longer hours was the most effective way they could deal with the sense of being overwhelmed, to the detriment of a work-life balance. Strategies which the GPs used to mitigate work stress included meditation, mindfulness, stress management techniques, taking regular exercise, eating well and meeting and debriefing with their colleagues, however the study also reported that a surprisingly high number of participants had adopted changes such as reducing their

For peer review only

working hours or changing their role to locum to allow them to downsize and regain work-life balance.(24)

Emotional hardening and cynicism among GPs appeared ubiquitous. An example of why this may be was provided by one GP who described going to identify a body after a suicide and on their return to a busy day and a list of patients, "*no-one even asked*" how they were, suggesting a resigned fatalism for at least one GP in this sample. Perhaps exacerbating the self-neglect reported was that GPs were concerned about the professional repercussions that might accompany accessing support. GPs raised the issue of potentially being declared unfit to practise if formal help was sought, a finding supported by previous research. Spiers et al.(25) found that GPs reported issues around privacy, confidentiality and the stigma surrounding seeking help. This worry around professional repercussions begins with medical training and is reported by medical students.(26)

The lack of uptake of services for mental health issues in the wake of the loss resonates with low GP uptake of care when physically ill in a study reported by Thompson and colleagues.(23) GPs want help but don't feel they can access it. The impact of this inability to seek help is seen in studies conducted on the mental health of doctors. Doctors experience high rates of anxiety,(27) depression,(28) substance misuse,(29) emotional exhaustion and burnout(30) and are at high risk of suicide themselves.(26) Male doctors are 1-4 times more likely than other men to die by suicide and female doctors 2-3 times more likely than other women.(31) This study showed that the impact of loses of this nature may even lead a GP to consider leaving the profession which has implications on personal, practice and regional service provision levels.

For peer review only - http://bmjopen.bmj.com/site/about/guidelines.xhtml

435 GP uncertainty about the availability of support may indicate their own ambivalence about help-seeking or the inappropriateness of current available support. While GPs are seen as part of the National Health Service in the UK, they are individual practitioners who work as self-employed practitioners within GP surgeries. One review of the literature recommended that doctors require specialist services which focus on the unique and complex situation of a doctor becoming a patient.(32) George et al.(33) outlined a pilot aimed at preventing or minimising mental health issues in doctors. This strategy would operate at a primary, secondary and tertiary level. Primary level prevention would be aimed mainly at medical students and may work to counteract the macho culture in which a doctor is expected to show no weakness. This level would include awareness-raising, enhancing coping skills and stress management skills. Secondary level prevention would allow early diagnosis and intervention and would include measures for screening, reducing stigma and creating a supportive culture for doctors. The tertiary level would include offering treatments and interventions to minimise the chronic negative effects of mental ill health on doctors.

Strengths of this study lie in the qualitative methodology employed in an area where depth of experience is complicated, nuanced and important to understand. The study allowed GPs to talk in depth about a subject matter which is both stigmatised and sombre. This allowed responses to be accessed in an area not widely researched in a population not comfortable discussing this topic. Findings showed that GPs are not comfortable but nevertheless need to talk about this loss, and this study provided a forum for them to do that, one which would not normally be presented. Limitations include that findings cannot necessarily be extrapolated to other medical settings outside of primary care and are not representative worldwide as care systems vary and different systems will offer different responses. However there is bound to be something fundamentally similar in the reaction to this loss from someone who has

For peer review only - http://bmjopen.bmj.com/site/about/guidelines.xhtml

dedicated their life to helping others. To know rather than surmise, further GPs, psychiatrists, psychologists and other suicide professionals should be incorporated in future research to offer additional insight and understanding. It is important to note also that the emotional aspect of guilt and self-scrutiny following the death of a patient by suicide may have contributed to potential under-reporting on the effects of patient suicide. Only by making the changes needed which will reduce the stigma around this issue can we know the scale as well as the nature of the impacts.

Conclusions

The findings of this study suggest a need to provide GPs with the appropriate psychological support after losing a patient to suicide. Despite a wish to remain true to the GP self-image of solid imperturbability created by adherence to the 'hidden contract', GPs report that the loss of a patient to suicide results in a period of self-scrutiny. From this may come short or longer term effects on their mental health. The "*hidden contract*" translates into doctors being unable to comfortably make a transition to patient which means that doctors are unable to properly receive the care they seek to provide for others.

Creating a culture shift which allows doctors to complete this transition will help prevent burnout and early retirement from a system which is not currently supporting them. One way to help create that change is to include an opposing view as part of training for all health and social care professionals for example by including in the curriculum modules on how to understand and deal with the effects of self-harm and suicide. The price for seeking that help should not include that their fitness to practice will be questioned consequently. GPs work in a high risk, high stress environment in which they are likely to experience loss which will require intervention and support. We recommend the provision of a strategy which will

For peer review only

facilitate the culture change needed and implement the services needed to support our doctors in general practice, including guidelines for GPs on when and how to ask for help. If a change is not facilitated, we will continue to see GPs experiencing distress and mental health issues which when unaddressed are contributing to mental illness, burnout and early retirement.

Contributors GMcA, KN and GL were responsible for data analysis and interpretations. GMcA wrote the initial draft of the article. GMcA revised the article in response to the comments of the co-authors after they commented critically on the draft. All authors gave their final approval of the version to be published.

Competing interests None declared.

Funding This study was funded by the Northern Ireland Public Health Agency (Grant COM/4032/08). The funder played no part in the conduct or reporting of the study, nor had any involvement in the interpretation of the data.

Data sharing statement No additional data available.

Figure 1: Thematic map showing relationship between themes

For peer review only

References

1. Leavey, G., Rosato, M., Galway, K., Hughes, L., Mallon, S., & Rondon, J. (2016). Patterns and predictors of help-seeking contacts with health services and general practitioner detection of suicidality prior to suicide: a cohort analysis of suicides occurring over a two-year period. *BMC psychiatry*, *16*, 1-8.

2. Sandford, D. M., Kirtley, O. J., Thwaites, R., & O'Connor, R. C. (2020). The impact on mental health practitioners of the death of a patient by suicide: a systematic review. In *Clinical Psychology & Psychotherapy* (Vol. 2). https://doi.org/10.1002/cpp.2515

3. Leavey, G., Mallon, S., Rondon-Sulbaran, J., Galway, K., Rosato, M., & Hughes, L. (2017). The failure of suicide prevention in primary care: family and GP perspectives–a qualitative study. *BMC psychiatry*, *17*(1), 1-10.

4. Pavlič, D. R., Treven, M., Maksuti, A., Švab, I., & Grad, O. (2018). General practitioners' needs for support after the suicide of patient: A qualitative study. *European Journal of General Practice*, *24*(1), 177–182. https://doi.org/10.1080/13814788.2018.1485648

5. Saini, P., Chantler, K., While, D., & Kapur, N. (2016). Do GPs want or need formal support following a patient suicide?: A mixed methods study. *Family Practice*, *33*(4), 414–420. https://doi.org/10.1093/fampra/cmw040

6. Alexander, D. A., Klein, S., Gray, N. M., Dewar, I. G., & Eagles, J. M. (2000). Suicide by patients: Questionnaire study of its effect on consultant psychiatrists. *British Medical Journal*, *320*(7249), 1571–1574. https://doi.org/10.1136/bmj.320.7249.1571

7. Murphy, P. T., Clogher, L., Van Laar, A., O'Regan, R., McManus, S., McIntyre, A., O'Connell, A., Geraghty, M., Henry, G., & Hallahan, B. (2019). The impact of service

For peer review only - http://bmjopen.bmj.com/site/about/guidelines.xhtml

user's suicide on mental health professionals. *Irish Journal of Psychological Medicine*, *April 2020*. https://doi.org/10.1017/ipm.2019.4

8. Finlayson, M., & Graetz Simmonds, J. (2018). Impact of Client Suicide on Psychologists in Australia. *Australian Psychologist*, *53*(1), 23–32. https://doi.org/10.1111/ap.12240
9. Gibbons, R., Brand, F., Carbonnier, A., Croft, A., Lascelles, K., Wolfart, G., & Hawton, K. (2019). Effects of patient suicide on psychiatrists: survey of experiences and support required. *BJPsych Bulletin*, *43*(5), 236–241. https://doi.org/10.1192/bjb.2019.26
10. Gaffney, P., Russell, V., Collins, K., Bergin, A., Halligan, P., Carey, C., & Coyle, S. (2009). Impact of patient suicide on front-line staff in Ireland. *Death Studies*, *33*, 639-656.
11. Foley, S. R., & Kelly, B. D. (2007). When a patient dies by suicide: Incidence, implications and coping strategies. *Advances in Psychiatric Treatment*, *13*(2), 134–138. https://doi.org/10.1192/apt.bp.106.002501
12. Zambrano, S. C., & Barton, C. A. (2011). On the journey with the dying: how general practitioners experience the death of their patients. *Death Studies*, *35*(9), 824–851.
13. Foggin, E., McDonnell, S., Cordingley, L., Kapur, N., Shaw, J., & Chew-Graham, C. A. (2016). GPs' experiences of dealing with parents bereaved by suicide: A qualitative study. *British Journal of General Practice*, *66*(651), e737–e746. https://doi.org/10.3399/bjgp16X686605
14. Halligan, P., & Corcoran, P. (2001). The impact of patient suicide on rural general practitioners. *British Journal of General Practice*, *51*(465), 295–296.

For peer review only - http://bmjopen.bmj.com/site/about/guidelines.xhtml

15. Leavey, G., Galway, K., Hughes, L., Mallon, S., Sulbarán, J. R., & Rosato, M. (2016). Understanding Suicide and Help-Seeking in Urban and Rural Areas in Northern Ireland. *Bamford Centre for Mental Health and Wellbeing, Ulster University: Belfast, UK*.

16. Braun, V., & Clarke, V. (2006). Using thematic analysis in psychology. *Qualitative research in psychology*, *3*, 77-101.

17. Smith, J. A., Flowers, P., & Larkin, M. (2013). *Interpretative Phenomenological Analysis: Theory, Method and Research. London*: SAGE Publications. https://doi.org/10.1080/14780880903340091

18. Larkin, M., Watts, S., & Clifton, E. (2006). Giving voice and making sense in interpretative phenomenological analysis. Qualitative Research in Psychology, 3(2), 102-120. Retrieved from ttps://www.tandfonline.com/doi/abs/10.1191/1478088706qp062oa

19. Ormston, R., Spencer, L., Barnard, M., & Snape, D. (2014). The foundations of qualitative research. *Qualitative research practice: A guide for social science students and researchers*, *2*, 52-55. Retrieved from http://www.jbposgrado.org/icuali/Qualitative%20Research%20practice.pdf

20. Savin-Baden, M., & Major, C. H. (2013). Qualitative research: The essential guide to theory and practice. https://www.routledge.com/Qualitative-Research-The-essential-guide-to-theory-and-practice-1st-Edition/Savin-Baden-Howell-Major/p/book/9780415674782.

For peer review only - http://bmjopen.bmj.com/site/about/guidelines.xhtml

21. O'Brien, B. C., Harris, I. B., Beckman, T. J., Reed, D. A., & Cook, D. A. (2014). Standards for reporting qualitative research: a synthesis of recommendations. *Academic medicine*, *89*(9), 1245-1251.

22. Leavey, G. (2008). UK clergy and people in mental distress: Community and patterns of pastoral care. *Transcultural Psychiatry*, *45*, 79-104.

23. Thompson, W. T., Cupples, M. E., Sibbett, C. H., Skan, D. I., & Bradley, T. (2001). Challenge of culture, conscience, and contract to general practitioners' care of their own health: qualitative study. *BMJ: British Medical Journal*, *323*(7315), 728.

24. Cheshire, A., Ridge, D., Hughes, J., Peters, D., Panagioti, M., Simon, C., & Lewith, G. (2017). Influences on GP coping and resilience: a qualitative study in primary care. *British Journal of General Practice*, *67*, e428-e436.

25. Spiers, J., Buszewicz, M., Chew-Graham, C. A., Gerada, C., Kessler, D., Leggett, N., ... & Riley, R. (2017). Barriers, facilitators, and survival strategies for GPs seeking treatment for distress: a qualitative study. *British Journal of General Practice*, *67*, e700-e708.

26. Devi, S. (2011). Doctors in distress. *The Lancet*, *377*(9764), 454-455.

27. Kroenke, K., Spitzer, R. L., Williams, J. B., Monahan, P. O., & Löwe, B. (2007). Anxiety disorders in primary care: prevalence, impairment, comorbidity, and detection. *Annals of internal medicine*, *146*, 317-325.

28. Firth-Cozens, J. (2001). Interventions to improve physicians' well-being and patient care. *Social science & medicine*, *52*, 215-222.

For peer review only - http://bmjopen.bmj.com/site/about/guidelines.xhtml

29. Bennett, J., & O'Donovan, D. (2001). Substance misuse by doctors, nurses and other healthcare workers. *Current opinion in Psychiatry*, *14*, 195-199.

30. Rø, K. E. I., Gude, T., Tyssen, R., & Aasland, O. G. (2008). Counselling for burnout in Norwegian doctors: one year cohort study. *BMJ*, *337*.

31. Schernhammer, E. S., & Colditz, G. A. (2004). Suicide rates among physicians: a quantitative and gender assessment (meta-analysis). *American Journal of Psychiatry*, *161*, 2295-2302.

32. Brooks, S. K., Gerada, C., & Chalder, T. (2011). Review of literature on the mental health of doctors: are specialist services needed?. *Journal of Mental Health*, *20*, 146-156.

33. George, S., Deshpande, S., & Kallivayalil, R. A. (2020). Are specialist treatment services needed for doctors with mental health problems?.*Indian Journal of Psychological medicine*, *42*, 105-107.

For peer review only

For peer review only - http://bmjopen.bmj.com/site/about/guidelines.xhtml

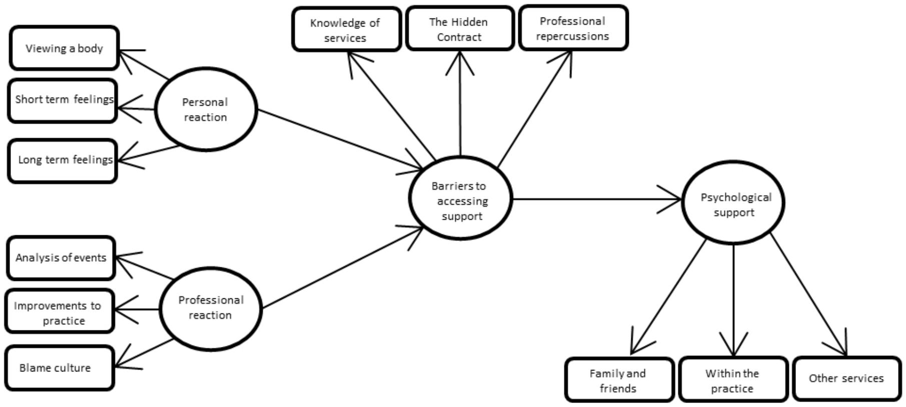

90x90mm (300 x 300 DPI)

Standards for Reporting Qualitative Research (SRQR)*

http://www.equator-network.org/reporting-guidelines/srqr/

Page/line no(s).

Title and abstract

**Title** - Concise description of the nature and topic of the study Identifying the study as qualitative or indicating the approach (e.g., ethnography, grounded theory) or data collection methods (e.g., interview, focus group) is recommended	Title Page Lines 1&2
**Abstract** - Summary of key elements of the study using the abstract format of the intended publication; typically includes background, purpose, methods, results, and conclusions	Page 1 Lines 1-16

Introduction

**Problem formulation** - Description and significance of the problem/phenomenon studied; review of relevant theory and empirical work; problem statement	Pages 3-4 lines 32-68
**Purpose or research questio**n - Purpose of the study and specific objectives or questions	Page 4 lines 60-68

Methods

**Qualitative approach and research paradigm** - Qualitative approach (e.g., ethnography, grounded theory, case study, phenomenology, narrative research) and guiding theory if appropriate; identifying the research paradigm (e.g., postpositivist, constructivist/ interpretivist) is also recommended; rationale**	Page 4/5 lines 71-81
**Researcher characteristics and reflexivity** - Researchers' characteristics that may influence the research, including personal attributes, qualifications/experience, relationship with participants, assumptions, and/or presuppositions; potential or actual interaction between researchers' characteristics and the research questions, approach, methods, results, and/or transferability	Page 6/7 lines 108-119
**Context** - Setting/site and salient contextual factors; rationale**	Page 5 lines 88-97
**Sampling strategy** - How and why research participants, documents, or events were selected; criteria for deciding when no further sampling was necessary (e.g., sampling saturation); rationale**	Page 5 lines 88-97
**Ethical issues pertaining to human subjects** - Documentation of approval by an appropriate ethics review board and participant consent, or explanation for lack thereof; other confidentiality and data security issues	Page 5 lines 72-81
**Data collection methods** - Types of data collected; details of data collection procedures including (as appropriate) start and stop dates of data collection and analysis, iterative process, triangulation of sources/methods, and modification of procedures in response to evolving study findings; rationale**	Page 5 lines 88-97<br>Page 6 lines 99-106

For peer review only - http://bmjopen.bmj.com/site/about/guidelines.xhtml

**Data collection instruments and technologies** - Description of instruments (e.g., interview guides, questionnaires) and devices (e.g., audio recorders) used for data collection; if/how the instrument(s) changed over the course of the study	Pages 5/6 lines 88-97
**Units of study** - Number and relevant characteristics of participants, documents, or events included in the study; level of participation (could be reported in results)	Page 7 lines 132-136
**Data processing** - Methods for processing data prior to and during analysis, including transcription, data entry, data management and security, verification of data integrity, data coding, and anonymization/de-identification of excerpts	Page 6 Lines 99-75
**Data analysis** - Process by which inferences, themes, etc., were identified and developed, including the researchers involved in data analysis; usually references a specific paradigm or approach; rationale**	Page 5 lines 93-101
**Techniques to enhance trustworthiness** - Techniques to enhance trustworthiness and credibility of data analysis (e.g., member checking, audit trail, triangulation); rationale**	Page 7 Lines 120-129

Results/findings

**Synthesis and interpretation** - Main findings (e.g., interpretations, inferences, and themes); might include development of a theory or model, or integration with prior research or theory	Pages 7-17 Lines 132- 363
**Links to empirical data** - Evidence (e.g., quotes, field notes, text excerpts, photographs) to substantiate analytic findings	Pages 7-17 Lines 132- 363

Discussion

**Integration with prior work, implications, transferability, and contribution(s) to the field -** Short summary of main findings; explanation of how findings and conclusions connect to, support, elaborate on, or challenge conclusions of earlier scholarship; discussion of scope of application/generalizability; identification of unique contribution(s) to scholarship in a discipline or field	Pages 18-22 Lines 364-445
**Limitations** - Trustworthiness and limitations of findings	Page 1/2 Lines 17-29<br>Page 22 Lines 446-462

Other

**Conflicts of interest** - Potential sources of influence or perceived influence on study conduct and conclusions; how these were managed	Page 24 line 490
**Funding** - Sources of funding and other support; role of funders in data collection, interpretation, and reporting	Page 24 Lines 491-493

*The authors created the SRQR by searching the literature to identify guidelines, reporting standards, and critical appraisal criteria for qualitative research; reviewing the reference lists of retrieved sources; and contacting experts to gain feedback. The SRQR aims to improve the transparency of all aspects of qualitative research by providing clear standards for reporting qualitative research.

For peer review only

For peer review only - http://bmjopen.bmj.com/site/about/guidelines.xhtml

**The rationale should briefly discuss the justification for choosing that theory, approach, method, or technique rather than other options available, the assumptions and limitations implicit in those choices, and how those choices influence study conclusions and transferability. As appropriate, the rationale for several items might be discussed together.

Reference:

O'Brien BC, Harris IB, Beckman TJ, Reed DA, Cook DA. **Standards for reporting qualitative research: a synthesis of recommendations.** *Academic Medicine*, Vol. 89, No. 9 / Sept 2014 DOI: 10.1097/ACM.0000000000000388

For peer review only
